# Ecological strategies determine continuous cropping susceptibility in *Panax* and *Achyranthes*

**DOI:** 10.3389/fpls.2026.1791596

**Published:** 2026-04-14

**Authors:** Meiling Yang, Mingming Wan, Limin Yang

**Affiliations:** Cultivation Base of State Key Laboratory for Ecological Restoration and Ecosystem Management, College of Traditional Chinese Medicine, Jilin Agricultural University, Changchun, China

**Keywords:** *Achyranthes bidentata*, continuous cropping problem, ecological strategy, *Panax* species, rhizosphere microbiome

## Abstract

**Introduction:**

Medicinal plants frequently suffer from severe continuous cropping problems, yet it remains unclear whether divergent ecological strategies underlie species-specific susceptibility to this problem.

**Methods:**

In this study, we investigated rhizosphere assembly patterns in three continuous cropping-sensitive *Panax* species (P. *ginseng*, P. *quinquefolius*, P. *notoginseng*) and the resilient species *Achyranthes bidentata* by analyzing paired cultivated and uncultivated soil samples from four major production regions in China. We measured soil physicochemical properties and enzyme activities and characterized bacterial (16S) and fungal (ITS) communities via amplicon sequencing. Plant-specific effects were quantified using Log_2_ fold change relative to uncultivated controls.

**Results:**

The *Panax* species aggressively remodeled their rhizosphere, inducing significant acidification and ammonium accumulation (Log_2_ FC up to 1.64 in P. *notoginseng*) while suppressing nitrification enzymes, and assembled fungal-dominated microbiomes enriched with pathogenic Nectriaceae, including *Ilyonectria* (LDA = 4.2) and *Neocosmospora* (LDA = 5.3). Their co-occurrence networks showed reduced stability, with negative correlations as low as 3.2%, and functional prediction indicated activated terpenoid metabolism (+74.8%). In contrast, A. *bidentata* maintained a neutral pH while specifically increasing available phosphorus (Log_2_ FC = +1.74) and nitrate nitrogen (Log_2_ FC = +0.74), and it enriched beneficial Actinobacteria by 15-85% and Hypocreales fungi. Its networks retained structural stability, with negative correlations of 12.7–18.6%. Plant species explained 60.5% of bacterial and 46.2% of fungal community variation, overwhelmingly exceeding the effect of soil compartment.

**Conclusion:**

We conclude that *Panax* employs a resource-acquisitive strategy that assembles unstable, pathogen-prone microbiomes, whereas A. *bidentata* adopts a resource-conservative strategy that fosters resilient communities. This ecological framework offers a predictive basis for developing tailored microbiome management in medicinal plant cultivation.

## 1 Introduction

Medicinal plants represent a valuable resource for both traditional medicine and modern pharmaceutical development. The rhizosphere microbiome, often described as a plant’s “second genome, “ critically influences medicinal quality and plant health by regulating nutrient cycling, plant immunity, and the synthesis of bioactive compounds (He et al., 2020; Huang et al., 2018). Understanding the assembly and function of this microbiome is therefore essential for advancing targeted ecological cultivation practices.

Driven by rising global demand, the intensive cultivation of Chinese medicinal herbs has led to increasingly severe continuous cropping problems (CCP) (Gao et al., 2006). The monoculture of high-value species such as *Panax ginseng* and *Panax notoginseng* frequently results in growth inhibition, disease outbreaks, and a marked decline in both yield and quality, which critically threatens the sustainability of the industry (Li et al., 2024; Luo et al., 2019). Accumulating evidence indicates that continuous cropping problems originate from disruptions to the rhizosphere micro-ecosystem (Qu et al., 2022). Under long-term monoculture, persistent selective pressure from root exudates degrades soil properties and shifts the microbial community from a beneficial to a pathogenic state. This shift enriches soil-borne pathogens while diminishing the function of beneficial microbes. Together, these changes establish a soil environment that suppresses the growth of the host plant (Behdarvandi and Goodwin, 2023; Li et al., 2024).

Soil physicochemical properties, enzyme activities, and microbial communities vary significantly with plant species and the specific root-associated compartment examined (Philippot et al., 2013). Distinct plant species shape their rhizosphere microenvironments through unique root exudation patterns, which in turn modify soil pH, nutrient availability, and enzyme activity profiles (Bais et al., 2006). Furthermore, within a single plant species, a distinct gradient exists from the rhizoplane to the rhizosphere soil and into the bulk soil, with each compartment representing a progressively weaker plant influence (Bais et al., 2006). Uncultivated soil provides a critical baseline, enabling the separation of plant-induced changes from the inherent heterogeneity of a site. A precise understanding of these compartment- and species-specific variations is therefore essential for assessing how medicinal plants modify their soil environment and recruit distinct microbial communities.

High-throughput sequencing has revealed microbial succession and pathogen enrichment in the CCP-affected soils of individual species, such as *Panax notoginseng* (Bao et al., 2022). However, these single-species studies cannot address whether medicinal plants with differing CCP susceptibility employ fundamentally different strategies to shape their rhizosphere microbiomes. This comparative knowledge gap limits our ability to predict which species are inherently vulnerable to CCP and why.

To address this gap, we apply plant ecological strategy theory (Bergmann et al., 2020; Reich, 2014) to develop a conceptual framework that distinguishes two contrasting modes of rhizosphere microbiome assembly. We hypothesize that plants employing a “resource-acquisitive” strategy rapidly modify rhizosphere chemistry via acidification and the enrichment of fast-cycling bacteria to maximize immediate nutrient uptake. This aggressive remodeling, however, may compromise microbial network stability and pathogen suppression. In contrast, plants with a “resource-conservative” strategy maintain a more neutral rhizosphere environment, which fosters diverse and stable microbial networks dominated by beneficial, slow-cycling taxa such as *Actinobacteria* to ensure long-term resilience. We further hypothesize that CCP-sensitive *Panax* species may employ an acquisitive strategy, whereas CCP-resilient A. *bidentata* may adopt a conservative one.

To test this framework, we selected model species representing contrasting susceptibilities to CCP: three highly sensitive *Panax* species (*Panax ginseng* C. A. Mey., *Panax quinquefolius* L., and *Panax notoginseng* (Burkill) F. H. Chen ex C. H. Chow) and one resilient species, *Achyranthes bidentata* Blume. *Panax* species are highly susceptible to CCP because their long cultivation cycles (3–6 years) (Yang et al., 2004), dense root systems that release substantial organic acids and allelopathic ginsenosides into the rhizosphere (Yang et al., 2017), and specific dependence on beneficial microbial associations (Wei et al., 2020) collectively intensify negative plant–soil feedback under continuous monoculture. In contrast, A. *bidentata* has shorter cultivation cycles (1–2 years), exudes roots with neutral polysaccharides and alkaloids that reduce soil acidification, and sustains more generalist microbial relationships, which together support its resilience in continuous cropping systems (Li et al., 2011).

This study aims to: (1) quantify species-specific modifications of rhizosphere physicochemical properties and enzyme activities to establish the chemical basis of each ecological strategy; (2) characterize the associated shifts in bacterial and fungal community structure, co-occurrence networks, and predicted functions to reveal the biological consequences of strategic divergence; and (3) synthesize these findings to test whether *Panax* species align with the hypothesized resource-acquisitive strategy while A. *bidentata* conforms to the resource-conservative strategy. We posit that this strategic divergence mechanistically explains species-specific CCP susceptibility and provides a scientific foundation for developing tailored microbiome management practices in sustainable medicinal plant cultivation.

## 2 Materials and methods

### 2.1 Sample collection

**Figure 1:**
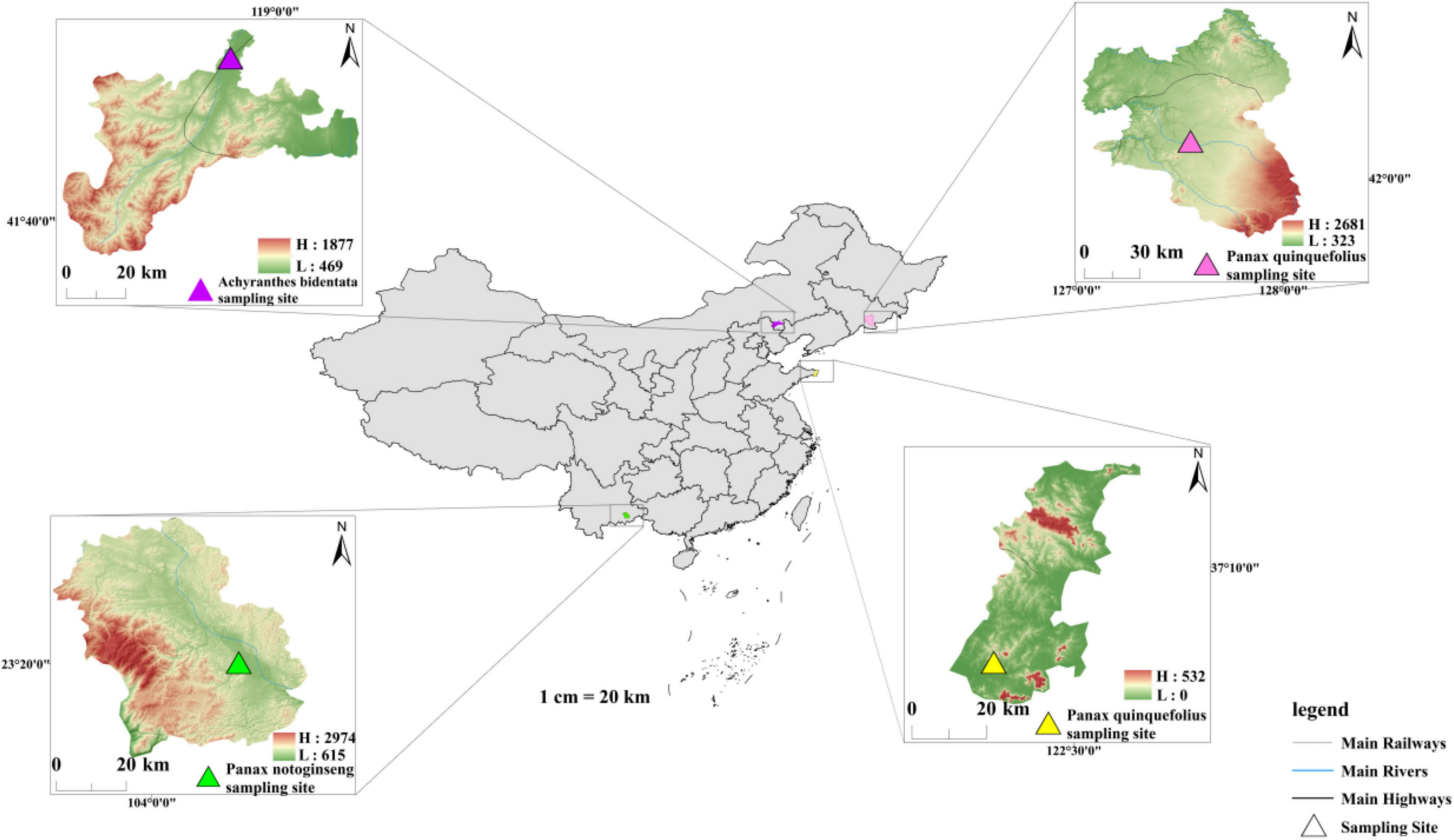
The map shows sampling sites in Fusong County (Jilin Province) for *Panax ginseng*, Rongcheng City (Shandong Province) for *Panax quinquefolius*, Wenshan Prefecture (Yunnan Province) for *Panax notoginseng*, and Harqin Banner (Inner Mongolia) for *Achyranthes bidentata*, spanning temperate to subtropical climatic zones. The base map of China was obtained from the National Platform for Common Geospatial Information Services (Tianditu), and county-level topographic shading was generated from GDEMV2 30M resolution digital elevation data sourced from the Geospatial Data Cloud.

Soil samples from the root zone of *Panax ginseng*, *Panax quinquefolius*, *Panax notoginseng*, and *Achyranthes bidentata* were collected in autumn of both 2022 and 2023 during their respective harvesting seasons. The sampling spanned temperate to subtropical climatic zones across China, from the northeast to Yunnan Province, covering four representative cultivation sites: Fusong County (Jilin Province) for P. *ginseng* (hereafter referred to as F6), Rongcheng City (Shandong Province) for P. *quinquefolius* (hereafter referred to as S4), Wenshan Prefecture (Yunnan Province) for P. *notoginseng* (hereafter referred to as Y3), and Harqin Banner (Chifeng, Inner Mongolia) for A. *bidentata* (hereafter referred to as C1). The sampling locations were mapped using ArcMap 10.8 (ESRI, USA) and are presented in **Figure 1**. Detailed sampling information is provided in **Supplementary Table 1**.

A standardized sampling protocol was applied in each cultivation field. The five-point sampling method was employed, deliberately avoiding depressions, mounds, and areas of abnormal plant growth. At each point, a sampling area of approximately 2 m² was delineated, typically covering two complete rows of medicinal plants. All healthy plants within this fixed area were carefully excavated, ensuring the root systems and adhering soil remained intact, using sterilized stainless steel shovels, spades, and forceps. To prevent cross-contamination, all tools were flame-sterilized with 95% ethanol between sampling points. To capture representative microbial community profiles while minimizing the influence of individual plant variation, soil from the 20–30 plants at each sampling point was composited and thoroughly homogenized to form a single biological replicate. This method of generating composite samples is standard in soil microbial ecology for assessing site-level trends (Edwards et al., 2015; Moinet et al., 2018). Adjacent, undisturbed uncultivated soil was collected in the same manner to serve as a paired control for each field. This approach isolated the directional change attributable to the plant itself by comparing cultivated soils to their corresponding uncultivated baselines.

Following this protocol across all sites and plant species, we obtained 80 final, homogenized soil samples from an initial collection of over 500 individual plants. This total comprises samples from four compartments, including rhizoplane soil (SS), rhizosphere soil (RS), bulk soil (BS), and uncultivated control (CK) for each of the four plant species, with five independent biological replicates per compartment-species combination (4 plants × 4 compartments × 5 replicates = 80 samples). All collected root-soil complexes were immediately placed in sterile bags, transported to the laboratory on ice, and subsequently processed to separate distinct root-associated compartments as described in Section 2.2.

### 2.2 Sample processing

Upon returning to the laboratory, each composite soil sample, representing one of the 80 unique sampling points, was processed to separate the distinct root-associated compartments. Bulk soil (BS) was collected by gently shaking the root system within a laminar flow cabinet. Rhizosphere soil (RS), defined as the soil adhering within 2 mm of the root surface, was obtained by brushing the roots (Moinet et al., 2018). The rhizoplane soil (SS) was subsequently isolated by subjecting the roots to ultrasonic treatment in PBS buffer for 30 s at 50–60 Hz, followed by centrifugation at 8, 000 rpm for 20 minutes at 4 °C (Edwards et al., 2015). All processed soil samples—SS, RS, BS, and the uncultivated control (CK)—were sieved through a 20-mesh screen to remove plant debris and other impurities.

Following compartment separation and sieving, all 80 samples were homogenized once more. Five representative aliquots were then prepared from each homogenized sample for parallel analysis. One part of each aliquot was flash-frozen in liquid nitrogen and stored at -80 °C for later 16S and ITS amplicon sequencing. The remainder was air-dried for assessing soil physicochemical properties and enzyme activities. The complete naming scheme for all experimental groups is detailed in **Supplementary Table 2**.

### 2.3 Analysis of soil physicochemical properties and enzyme activities

This study determined 11 physicochemical properties and 7 soil enzyme activities in the rhizosphere soil (RS), bulk soil (BS), and uncultivated control soil (CK). Rhizoplane soil (SS) was not included in these analyses due to the limited sample quantity obtained after ultrasonic separation, which was sufficient only for DNA extraction and subsequent microbial community sequencing (Section 2.4). All physicochemical analyses followed standard methods (Bao, 2000). For physicochemical properties, we measured: water content (WC) by oven-drying at 105 °C; soil pH in a 1:2.5 (w/v) soil-water suspension; organic matter (OM) by potassium dichromate oxidation; total nitrogen (TN) by the Kjeldahl method; total phosphorus (TP) by molybdenum antimony colorimetry after H_2_SO_4_-HClO_4_ digestion; total potassium (TK) by flame photometry after NaOH melting; available phosphorus (AP) by NaHCO_3_ extraction and colorimetry; available potassium (AK) by NH_4_OAc extraction and flame photometry.

For nitrogen fractions, three complementary measurements were conducted to assess both overall nitrogen supply and specific transformation processes. Available nitrogen (AN), also termed alkali-hydrolyzable nitrogen, was determined by the alkali hydrolysis diffusion method to evaluate the soil’s capacity to supply nitrogen over a growing season. In addition, inorganic nitrogen fractions—ammonium nitrogen (NH_4_^+^-N) and nitrate nitrogen (NO_3_^-^-N)—were separately measured to track nitrogen transformation dynamics. These two forms differ fundamentally: ammonium nitrogen is directly assimilated but requires energy for amino acid incorporation and is preferentially taken up under acidic conditions, while nitrate nitrogen is more mobile in soil solution, serves as both nutrient and signaling molecule, and its accumulation indicates active nitrification. Both inorganic fractions were extracted with 2 M KCl and determined by indophenol blue colorimetry for NH_4_^+^-N and phenol disulfonic acid colorimetry for NO_3_^-^-N, respectively. The balance between these forms reflects soil redox status and microbial nitrification activity, making their separate measurement essential for understanding nitrogen cycling in response to plant cultivation.

Enzyme activities were assayed as follows (Guan, 1986): Urease (S-URE) activity was determined by colorimetry of ammonium nitrogen released after incubation with urea. Acid phosphatase (S-ACP) activity was measured based on the release of *p*-nitrophenol from *p*-nitrophenyl phosphate. Sucrase (S-SUC) activity was assessed by the 3, 5-dinitrosalicylic acid method. Catalase (S-CAT) activity was quantified by titrating the residual H_2_O_2_ with KMnO_4_. The activities of β-glucosidase (S-β-GC), nitrate reductase (S-NAR), and nitrite reductase (S-NIR) were determined using specific commercial assay kits (Solaibao, China) according to the manufacturer’s instructions.

### 2.4 DNA extraction and amplicon sequencing

We conducted nucleic acid extraction and amplicon sequencing on soil samples from four compartments (SS, RS, BS, CK) across the four medicinal plant species.

Total genomic DNA was extracted from the samples using the cetyltrimethylammonium bromide (CTAB) method. The concentration and purity of the DNA were assessed by 1% agarose gel electrophoresis. Based on the quantification results, the DNA was diluted with sterile water to a working concentration of 1 ng/µL.

The V5-V7 hypervariable regions of the bacterial 16S rRNA gene were specifically amplified using barcoded primers 799F (5’-AACMGGATTAGATACCCKG-3’) and 1193R (5’-ACGTCATCCCCACCTTCC-3’) (Bodenhausen et al., 2013; Huang et al., 2020); The ITS1 region of the fungal ITS gene was amplified using the specific primers ITS1-1F (5’-CTTGGTCATTTAGAGGAAGTAA-3’) and ITS1-1R (5’-GCTGCGTTCTTCATCGATGC-3’) (Li et al., 2016; Xiong et al., 2017). All PCR reactions were performed in a 15 µL volume containing the Phusion^®^ High-Fidelity PCR Master Mix (New England Biolabs), 2 µM of each forward and reverse primer, and approximately 10 ng of template DNA. All primers were stored at -20 °C upon arrival, and working dilutions were maintained at 4 °C during experimental procedures. The thermal cycling protocol comprised an initial denaturation at 98 °C for 1 min, followed by 30 cycles of denaturation at 98 °C for 10 s, annealing at 50 °C for 30 s, and extension at 72 °C for 30 s.

PCR amplicons were mixed with an equal volume of 1× TAE buffer and visualized by electrophoresis on a 2% agarose gel. Products were then pooled in equimolar amounts and purified using the Universal DNA Purification Kit (Tianjin, China). Sequencing libraries were constructed with the NEB Next^®^ Ultra DNA Library Prep Kit (Illumina, USA) following the manufacturer’s instructions, with the addition of index codes. Library quality was assessed using the Agilent 5400 system (Agilent Technologies, USA), and final libraries were sequenced on the Illumina NovaSeq platform to generate 250-bp paired-end reads.

### 2.5 Sequence processing and taxonomic annotation

Microbial community analysis was performed using the QIIME2 pipeline (version 2019.1; https://docs.qiime2.org/2019.1/). Raw FASTQ sequences were first imported and subjected to quality control. Denoising, paired-end read merging, and chimera removal were conducted using the DADA2 plugin, resulting in high-quality amplicon sequence variants (ASVs) (Callahan et al., 2016). Representative sequences were then taxonomically classified using the feature-classifier plugin by aligning them against the Greengenes2 database (version 2022.10) for bacteria and the UNITE fungal database (version 2018.11) for fungi, respectively (Bokulich et al., 2018). Finally, sequences assigned to mitochondria and chloroplasts were removed. For fungal data, additional filtering was applied to exclude non-target eukaryotic sequences (e.g., from animals and plants), thereby ensuring the accuracy of subsequent analyses.

### 2.6 Acquisition of macro-climatic variables

To account for potential climatic influences on soil microbial communities, we extracted 11 bioclimatic variables for each sampling site based on GPS coordinates. Data were obtained from the WorldClim2 global climate database at a spatial resolution of 30 arc-seconds (Fick and Hijmans, 2017). The selected variables with their respective units include: Annual Mean Temperature (Bio1, °C), Mean Diurnal Range (Bio2, °C), Isothermality (Bio3, %), Temperature Seasonality (Bio4, °C *100), Max Temperature of Warmest Month (Bio5, °C), Min Temperature of Coldest Month (Bio6, °C), Temperature Annual Range (Bio7, °C), Annual Precipitation (Bio12, mm), Precipitation of Wettest Month (Bio13, mm), Precipitation of Driest Month (Bio14, mm), and Precipitation Seasonality (Bio15, %).

### 2.7 Statistical analysis

#### 2.7.1 Analysis of soil physicochemical properties and enzyme activities

Differences in soil physicochemical properties and enzyme activities among groups (defined by plant species and soil compartment) were analyzed using IBM SPSS Statistics 27. Data normality was assessed using the Shapiro-Wilk test (α = 0.05) supplemented by visual inspection of Q-Q plots, and homogeneity of variances was evaluated using Levene’s test. As significant heterogeneity of variances was detected across groups (*p* < 0.05), Welch’s analysis of variance was employed as a robust alternative to standard ANOVA for testing overall group mean differences. When Welch’s ANOVA indicated significant effects, *post-hoc* pairwise comparisons were conducted using the Games-Howell procedure, which does not assume equal variances or equal sample sizes. The significance threshold was set at *p* < 0.05, and 95% confidence intervals adjusted for multiple comparisons are reported to control the family-wise error rate. Data are presented as mean ± standard deviation.

#### 2.7.2 Multivariate and microbial community analyses

To explore overall differences among samples and quantify plant impacts on soil properties, principal component analysis was performed after Z-score standardization of climatic and soil factors using R version 4.4.3 (Cowan et al., 2022). Additionally, the log_2_ fold change (Log_2_ FC) for each parameter between cultivated and uncultivated soils was calculated as an effect size to evaluate species-specific rhizosphere effects.

#### 2.7.3 Microbial diversity and community structure analysis

For microbial data, alpha diversity indices including Chao1 and Shannon were calculated using QIIME2. Non-metric multidimensional scaling based on Bray-Curtis distance, together with Permutational Multivariate Analysis of Variance (PERMANOVA) based on Unifrac distance, was applied to assess within-sample species richness and between-group community structure differences (Vázquez-Baeza et al., 2013). Linear discriminant analysis effect size was employed to identify microbial biomarkers exhibiting significant differences between groups (Love et al., 2014).

#### 2.7.4 Environmental drivers and network analysis

Redundancy analysis was performed with the ‘vegan’ package to examine relationships between microorganisms and environmental factors (Dixon, 2003). A co-occurrence network of dominant taxa was constructed based on Spearman correlations (r > 0.8, *p* < 0.05), and its topological attributes were calculated in Gephi to reveal interspecific interaction patterns. Networks derived from correlation analyses identify statistical associations, which may reflect ecological interactions like competition or mutualism but can also stem from shared environmental preferences or indirect effects. Consequently, these patterns should be interpreted as hypotheses of potential interactions rather than as direct evidence of specific ecological processes.

#### 2.7.5 Microbial functional prediction

Based on 16S rRNA gene data and ITS data respectively, Phylogenetic Investigation of Communities by Reconstruction of Unobserved States 2 (PICRUSt2) for bacterial communities and the Metabolic Pathway Database (MetaCyc) database for fungal communities were employed to predict potential functional composition (Langille et al., 2013). It should be noted that these functional profiles are predictions inferred from marker gene data and should be considered exploratory.

## 3 Results

### 3.1 Species−specific patterns in the shaping of medicinal plant root zone microenvironments

**Figure 2:**
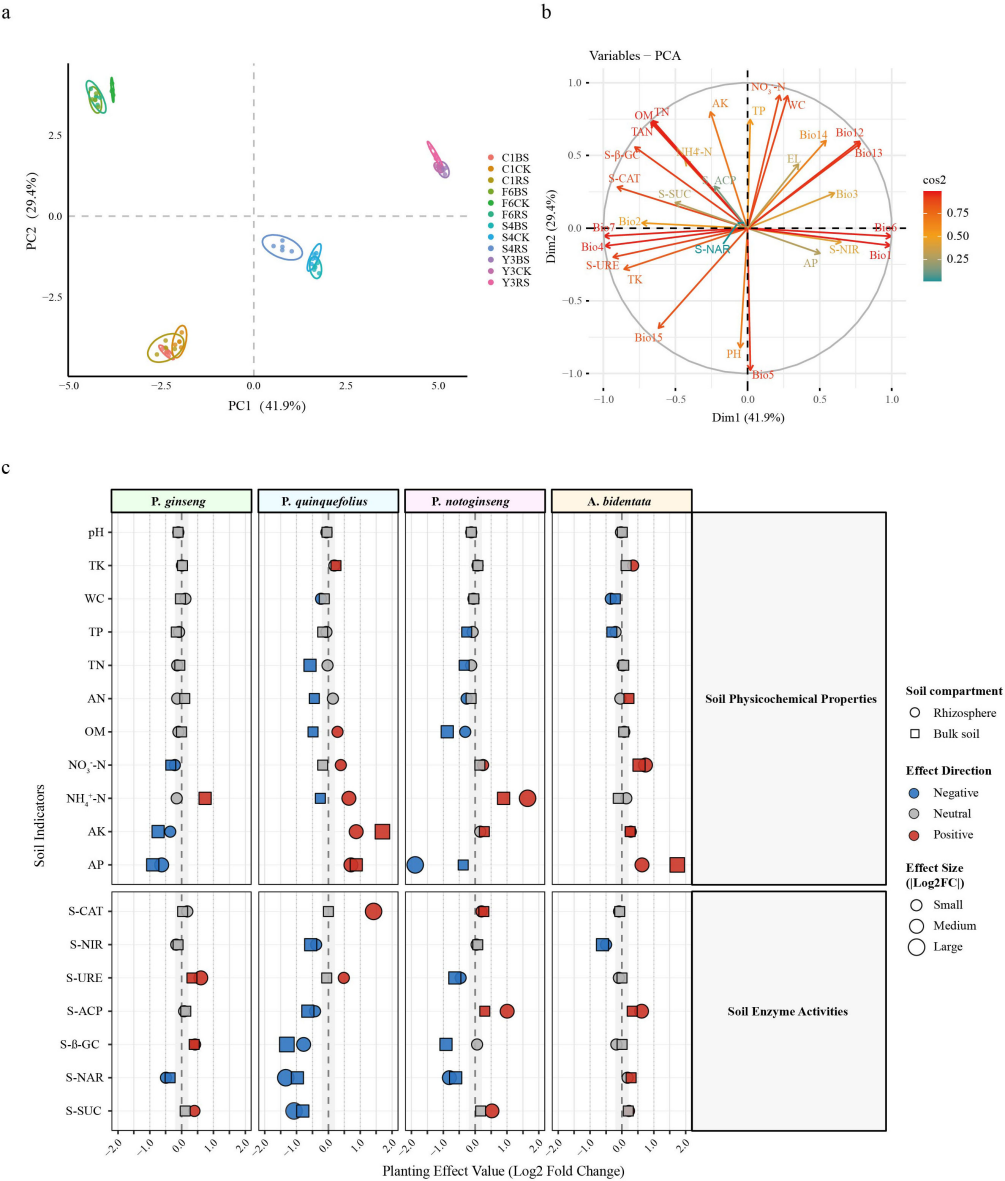
Principal component analysis and quantification of soil variable effects. **(a)** PCA score plot showing sample distribution based on soil physicochemical properties and climatic factors. Each point represents an individual biological replicate. Different colors represent the 12 sample groups consisting of four plant species and three soil compartments. Ellipses represent 95% confidence intervals for each species-compartment group. **(b)** Variable correlation circle plot displaying the contribution of each variable to the principal components. Arrow length indicates contribution strength; arrow direction indicates correlation patterns among variables. **(c)** Standardized effect sizes (Log_2_ fold change, Log_2_ FC) of cultivated soils relative to paired uncultivated controls. Point size indicates effect magnitude: small (|Log_2_ FC| < 0.5), medium (0.5 ≤ |Log_2_ FC| < 1.0), large (|Log_2_ FC| ≥ 1.0). Color indicates effect direction: red (positive), blue (negative), and gray (|Log_2_ FC| < 0.2, considered non-significant). Shape distinguishes soil compartments: circle (RS) and square (BS). The chart is faceted by variable type (physicochemical properties, enzyme activities) and plant species. Sample codes: F6 (P. *ginseng*), S4 (P. *quinquefolius*), Y3 (P. *notoginseng*), C1 (A. *bidentata*), followed by CK, BS, or RS.

Principal component analysis based on soil physicochemical properties, enzyme activities, and climatic factors revealed distinct clustering patterns among samples from different regions (**Figure 2A**). Samples from the P. *ginseng* cultivation site were primarily associated with higher organic matter and total nitrogen, while samples from the P. *notoginseng* site were closely linked to precipitation-related variables (Bio12, Bio13). The P. *quinquefolius* site showed associations with higher pH, and the A. *bidentata* site was primarily influenced by precipitation seasonality (Bio15) (**Figure 2B**). These regional differences in baseline soil properties and climatic conditions underscore the importance of our paired sampling design, which uses uncultivated controls to isolate plant-specific effects from inherent site heterogeneity.

To isolate plant-specific effects from regional heterogeneity in uncultivated soils (**Figures 2A, B**), we quantified the rhizosphere effect using the Log_2_ fold change (Log_2_FC) of bulk soil (BS) and rhizosphere soil (RS) relative to their paired uncultivated control (CK). The original physicochemical properties and enzyme activities, presented as mean ± SD, are detailed in **Supplementary Tables 3**, **4**, respectively.

The results revealed significant species-specific regulation of the root zone microenvironment by different medicinal plants (**Figure 2C**). *Panax* species exhibited systematic environmental remodeling characteristics: all three species significantly decreased soil pH and promoted NH_4_^+^-N accumulation, as exemplified by bulk soil of *P. ginseng* (F6BS, Log_2_ FC = +0.74) and rhizosphere soil of *P. notoginseng* (Y3RS, Log_2_ FC = +1.64), while reducing phosphorus content, notably total phosphorus in bulk soil of *P. quinquefolius* (S4BS, Log_2_ FC = -0.18) and available phosphorus in rhizosphere soil of *P. notoginseng* (Y3RS, Log_2_ FC = -1.88). Enzyme activity analysis indicated consistent inhibition of nitrate reductase (S-NAR) and nitrite reductase (S-NIR), reflecting their systematic regulation of the nitrogen transformation pathway.

In contrast, *Achyranthes bidentata* exhibited a highly specific and conservative root zone regulation strategy: while maintaining stable soil pH and most enzyme activities, it specifically promoted the enrichment of available phosphorus (AP Log_2_ FC = +1.74) in bulk soil (C1BS) and nitrate nitrogen (NO_3_^-^-N Log_2_ FC = +0.74) in rhizosphere soil (C1RS), with limited effects on other soil properties.

### 3.2 Microbial diversity reveals species-specific strategies in root zone microecological assembly

**Figure 3:**
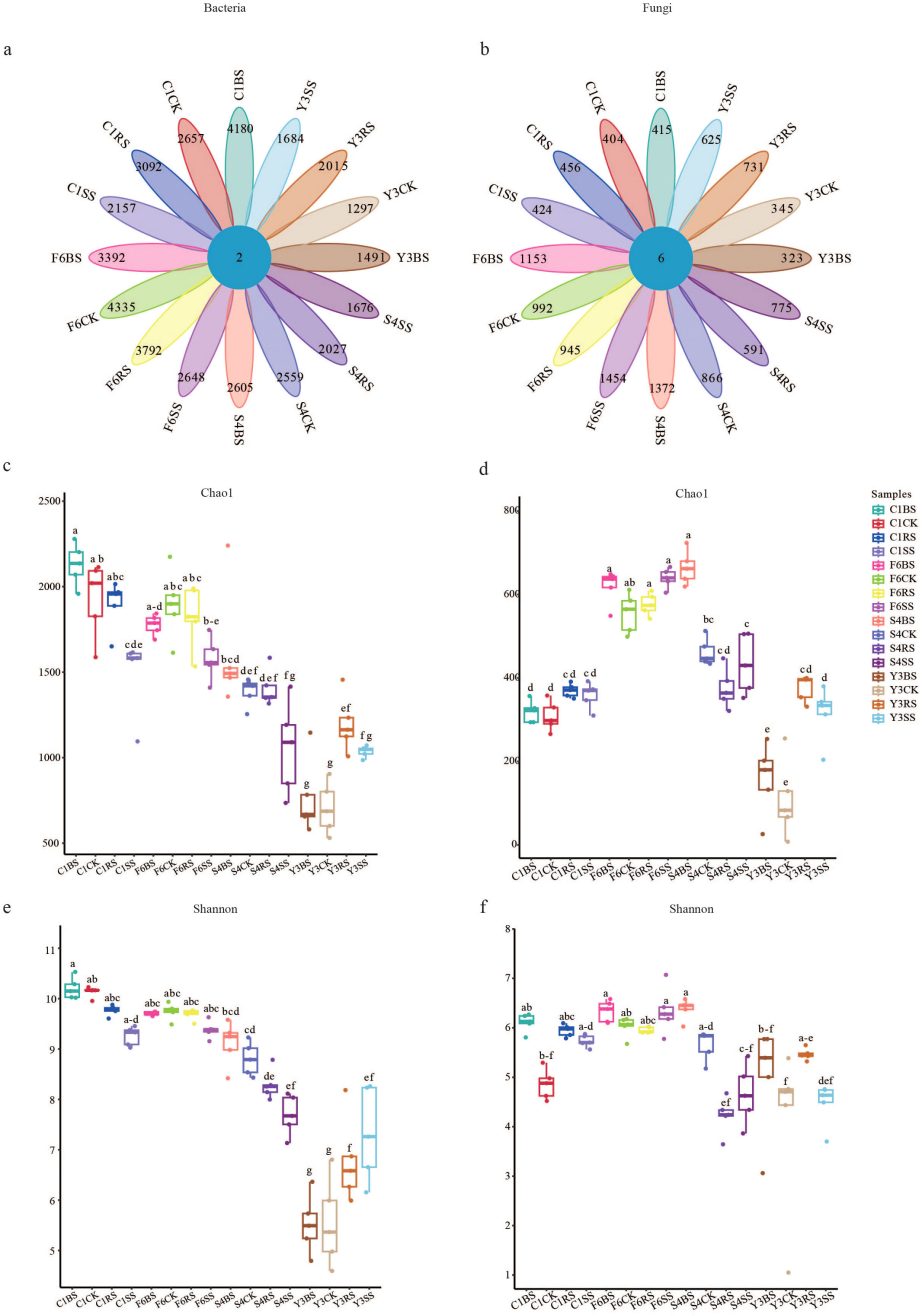
Variations in ASV numbers and α-diversity across soil sample groups. **(a, b)** Chord diagrams showing ASV numbers for bacterial **(a)** and fungal **(b)** communities. Each petal represents one soil compartment of one plant species, with ASV numbers displayed within each petal. Different colors distinguish the 16 species-compartment combinations: four plant species (F6: P *ginseng*; S4: P *quinquefolius*; Y3: P *notoginseng*; C1: A *bidentata*) × four soil compartments (CK, uncultivated control; BS, bulk soil; RS, rhizosphere soil; SS, rhizoplane soil). **(c–f)** α-diversity indices in different soil compartments: **(c)** bacterial Chao1 richness estimator, **(d)** fungal Chao1, **(e)** bacterial Shannon diversity index, **(F)** fungal Shannon. Boxplots show median, interquartile range, and outliers. Different lowercase letters indicate significant differences among groups (*p* < 0.05, Welch’s ANOVA with Games-Howell *post-hoc* test); shared letters denote no significant difference.

Based on high-throughput sequencing data from 80 soil samples, a total of 8, 510, 210 bacterial sequences and 82, 331, 824 fungal sequences were obtained, which were clustered into 51, 576 bacterial ASVs and 14, 701 fungal ASVs. Analysis indicated that although uncultivated soils exhibited significant geographical heterogeneity in microbial communities, plant cultivation exerted a marked species-specific influence on root zone microbial assemblages (**Figure 3**).

Based on the variation patterns of ASV counts, the four medicinal plants exhibited distinct regulatory modes (**Figures 3A, B**). *Panax notoginseng* demonstrated a comprehensive promoting effect, with significant increases in both bacterial and fungal ASV numbers in its rhizosphere (Y3RS) and rhizoplane soils (Y3SS). In contrast, *Panax quinquefolius* showed an overall inhibitory pattern. Meanwhile, *Panax ginseng* and *Achyranthes bidentata* displayed opposite “preferential” regulatory strategies. Specifically, *P. ginseng* cultivation significantly reduced bacterial ASV counts (with a decrease of up to 39.0% in rhizoplane soil, F6SS) but markedly increased fungal ASV numbers (with a net increase of 576 across all compartments). Conversely, *A. bidentata* cultivation significantly promoted bacterial ASV counts (with an increase of 57.4% in bulk soil, C1BS), while its promoting effect on fungal ASVs was relatively weak (total increase only 14.4% of that observed in *P. ginseng*).

Analysis of α-diversity (**Figures 3C–F**) further corroborated this trend. The bacterial Chao1 and Shannon indices were generally higher across all compartments of *Achyranthes bidentata* than in *Panax* species, whereas the latter maintained higher fungal diversity. Furthermore, significant divergence was observed among different compartments: microbial diversity in the bulk soil of *Panax quinquefolius* (S4BS) was higher than in uncultivated soil (S4CK), while it generally decreased in the rhizosphere(S4RS) and rhizoplane (S4SS). In contrast, *Panax notoginseng* showed significantly enhanced microbial diversity in the rhizosphere (Y3RS), but relatively lower bacterial diversity in its bulk soil (Y3BS).

### 3.3 Plant species identity governs root zone microbial community structure

**Figure 4:**
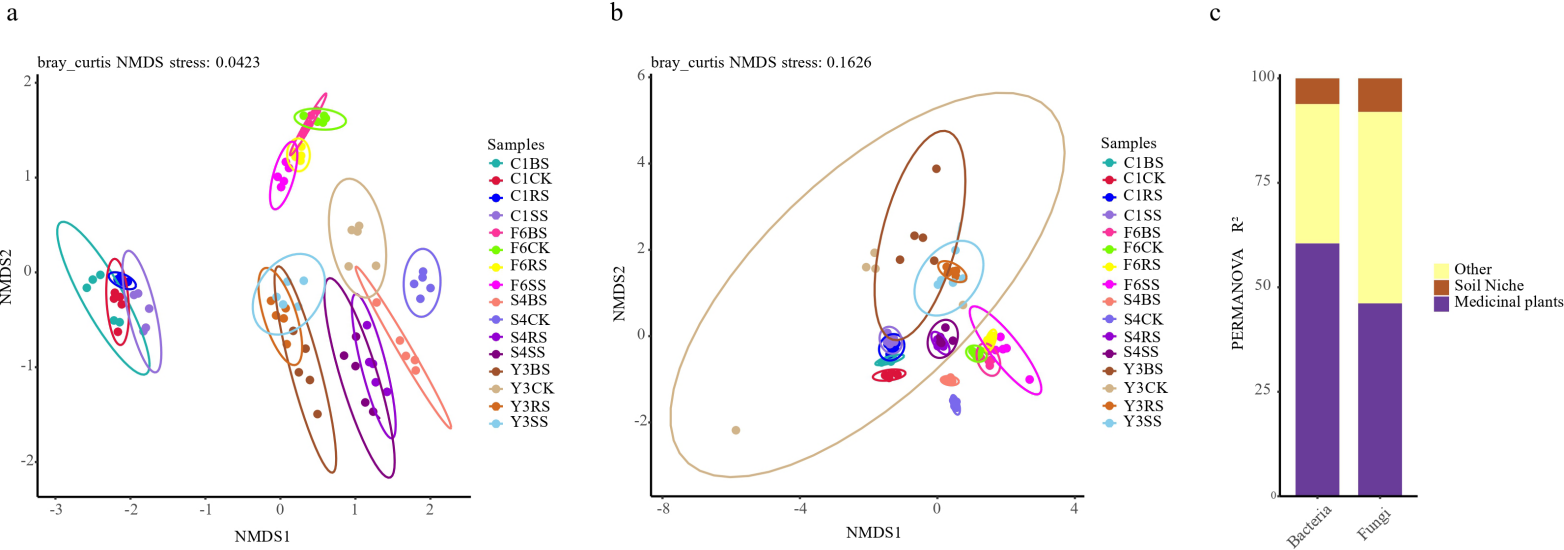
Microbial community structure in different soil compartments of medicinal plants. **(a, b)** Non-metric multidimensional scaling (NMDS) ordination based on Bray-Curtis distance showing bacterial **(a)** and fungal **(b)** community composition across all samples (n = 80). Each point represents an individual sample. Colors represent the 16 species-compartment combinations: four plant species (F6: P *ginseng*; S4: P *quinquefolius*; Y3: P *notoginseng*; C1: A. *bidentata*) × four soil compartments (CK, uncultivated control; BS, bulk soil; RS, rhizosphere soil; SS, rhizoplane soil). Stress values indicate goodness of fit: bacterial stress = 0.0423, fungal stress = 0.1626. **(c)** PERMANOVA results based on Bray-Curtis distance showing percentage contribution of plant species, soil compartment, their interaction, and residual factors to bacterial and fungal community variation. All factors were significant at *p* < 0.05.

To elucidate the assembly patterns of root zone microbial communities following medicinal plant cultivation, this study conducted non-metric multidimensional scaling (NMDS) and PERMANOVA based on all samples. The NMDS ordination based on Bray-Curtis distance (**Figures 4A, B**) revealed distinct species-specific clustering of samples. Notably, samples from all compartments of *Achyranthes bidentata* were clearly separated from those of *Panax* species (bacterial community stress = 0.042; fungal community stress = 0.163), whereas samples of the three *Panax* species clustered closely together, indicating that plant species is a key driver of microbial community assembly. This trend was further confirmed by NMDS analysis based on unweighted UniFrac distance (**Supplementary Figures 1A, B**), and comparison of inter-group distances more intuitively illustrated the extent of community differences between cultivated and uncultivated soils (**Supplementary Figures 1C–J**).

PERMANOVA analysis statistically confirmed the dominant role of plant species (**Supplementary Tables 6**, **7**). For bacterial communities, all cultivated soil compartments of *Panax* species showed significant separation from uncultivated soil (pseudo-F = 2.03-4.04, *p* < 0.01), whereas the degree of separation was relatively weaker in *Achyranthes bidentata* (pseudo-F = 1.19-2.84, *p* < 0.01). A similar pattern was observed in fungal communities, with *Panax* species exhibiting an overall stronger separation (pseudo-F = 1.86-5.12, *p* < 0.01) than *A. bidentata* (pseudo-F = 2.25-2.80, *p* < 0.01). Notably, Panax quinquefolius demonstrated the strongest community-shaping effect (pseudo-F = 4.09-5.12, *p* < 0.01).

Additionally, an intriguing finding was that microbial community structure exhibited a gradient pattern across different soil compartments, with community differences intensifying as spatial distance increased, indicating a gradient filtering effect of plants on root zone microorganisms.

To further quantify the contribution of each factor, we analyzed their effects on community variation (**Figure 4C**). The results demonstrated that “medicinal plant species” was the dominant factor driving microbial community assembly, explaining 60.5% and 46.2% of the variation in bacterial and fungal communities, respectively. This contribution was significantly higher than that of “soil compartment”. Together, the two factors and their interaction explained 66.7% and 54.3% of the total variation in bacterial and fungal communities, respectively, further underscoring the central role of plant species in shaping the root zone microenvironment.

### 3.4 Species-specific regulation of root zone microbial community composition by medicinal plants

To elucidate the regulatory effects of medicinal plants on the composition of the root zone microbial community, this study analyzed the microbial composition of different soil samples at the phylum and genus levels.

**Figure 5:**
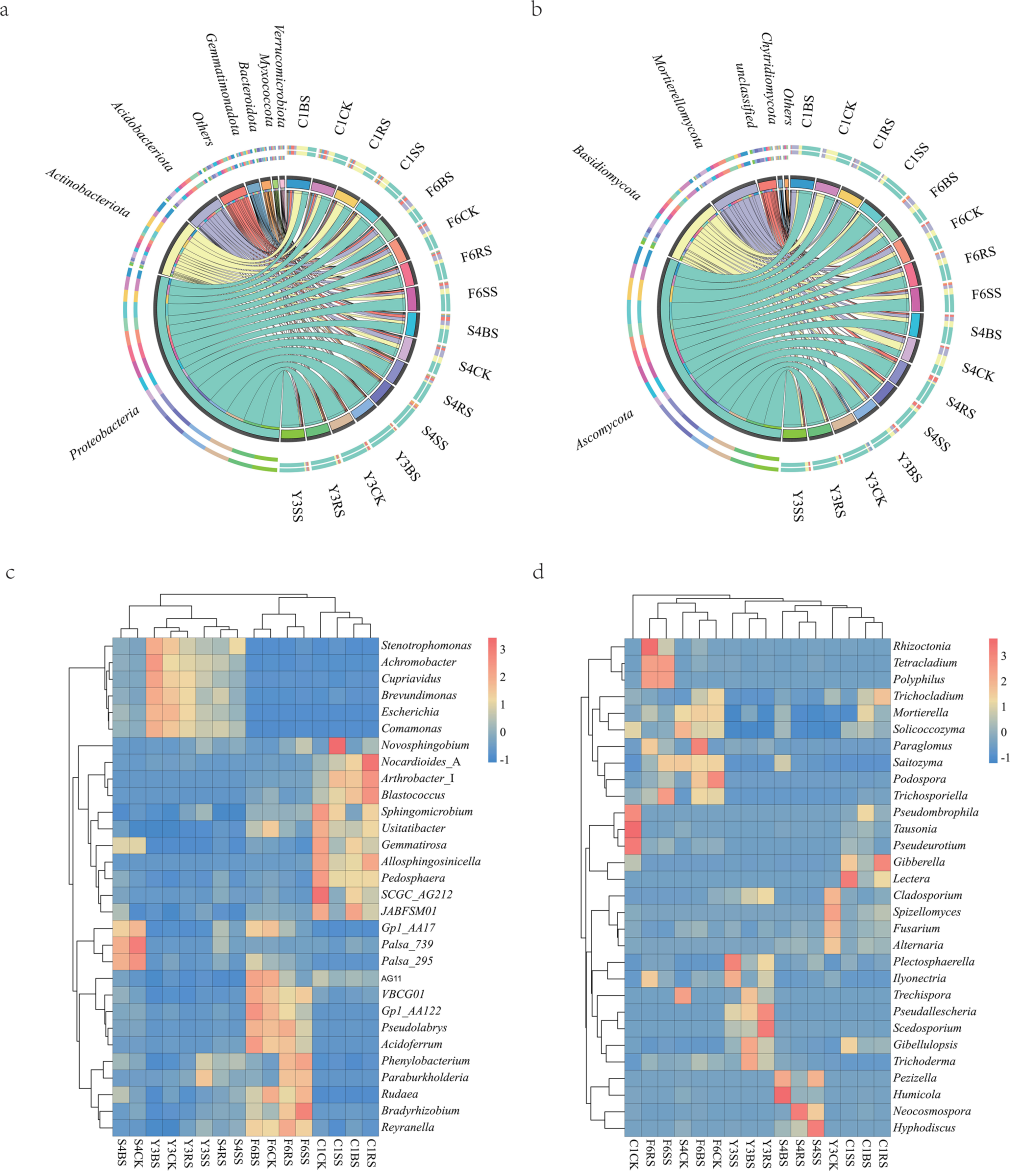
Composition of microbial communities in different soil compartments of medicinal plants. **(a, b)** Phylum-level community composition of bacterial **(a)** and fungal **(b)** communities. Each color represents a different phylum. Phyla with relative abundance below 1% in any sample are grouped into the “Others” category. Samples are grouped by plant species (F6: P *ginseng*; S4: P *quinquefolius*; Y3: P *notoginseng*; C1: A *bidentata*) and soil compartments (CK, uncultivated control; BS, bulk soil; RS, rhizosphere soil; SS, rhizoplane soil). **(c, d)** Heatmaps showing the top 30 most abundant bacterial **(c)** and fungal **(d)** genera. Color gradient from blue to red represents relative abundance (low to high). Rows represent genera; columns represent individual samples, with sample codes indicating plant species and soil compartments as in **(a, b)**. Uncultured candidate taxa (e.g., Palsa 739, Gp1 AA17) are displayed with their original identifiers.

At the phylum level, distinct microbial enrichment patterns were observed in the root zones of different medicinal plants (**Figures 5A, B**). *Achyranthes bidentata* exhibited consistent enrichment of *Actinobacteria*, with its relative abundance in bulk soil (C1BS), rhizosphere soil (C1RS), and rhizoplane soil (C1SS) increasing by 77.5%, 85.0%, and 15.0%, respectively, compared to uncultivated soil (C1CK). In contrast, *Panax* species displayed diversified regulatory patterns: *P. quinquefolius* significantly suppressed *Actinobacteria* (with an 81.8% decrease in rhizoplane soil, S4SS) while concurrently enriching *Proteobacteria* (relative abundance in S4SS was 2.05 times that in S4CK) and *Bacteroidetes* (3.3-fold increase in S4SS). Meanwhile, *P. notoginseng* enriched *Actinobacteria* (93.8% increase in rhizosphere soil, Y3RS) while substantially enhancing *Gemmatimonadetes* (1.2-2.0-fold increase across root zone compartments) and *Myxococcota* (2.0-fold increase in relative abundance in Y3RS) (**Supplementary Figure S2 A–G**).

Fungal community analysis further revealed plant-specific regulatory patterns (**Supplementary Figure S2 H–K**). The root zone of *Achyranthes bidentata* was significantly enriched with *Ascomycota* (67.6% increase in rhizoplane soil, C1SS), whereas *Panax notoginseng* specifically enriched *Basidiomycota* (relative abundance in Y3SS was 2.61 times that in Y3CK), forming a sharp contrast with the suppressed *Basidiomycota* in the root zone of *A. bidentata* (64.6%-84.3% decrease). Furthermore, *Mortierellomycota* was significantly enriched in the bulk soil of *A. bidentata* (C1BS, relative abundance 2.28 times that in C1CK) but consistently suppressed in the root zones of all *Panax* species (e.g., 90.7% decrease in the rhizoplane soil of *P. quinquefolius*, S4SS).

Analysis of the heatmap at the genus level (**Figures 5C, D**) showed that samples exhibited clear clustering trends according to plant species. The relative abundances of genera such as *Stenotrophomonas* and *Achromobacter* were significantly increased in all soil compartments of *Panax notoginseng*. In contrast, genera including *Pseudolabrys* and *Acidothermus* dominated the root zone of *Panax ginseng*, while the rhizosphere of *Achyranthes bidentata* (C1RS) was characterized by the widespread distribution of genera such as *Nocardioides* and *Arthrobacter*. Fungal communities also displayed significant structural differentiation: the bulk soil of *Panax ginseng* (F6BS) was relatively similar in community composition to uncultivated soil (F6CK), whereas the cultivated soils of *A. bidentata* and *P. notoginseng* formed distinct fungal community structures.

### 3.5 Root zone microbial biomarkers reveal species-specific recruitment patterns

To investigate the species-specific recruitment of root zone microorganisms by the four medicinal plants, this study identified microbial biomarkers in different soil compartments through LEfSe analysis.

**Figure 6:**
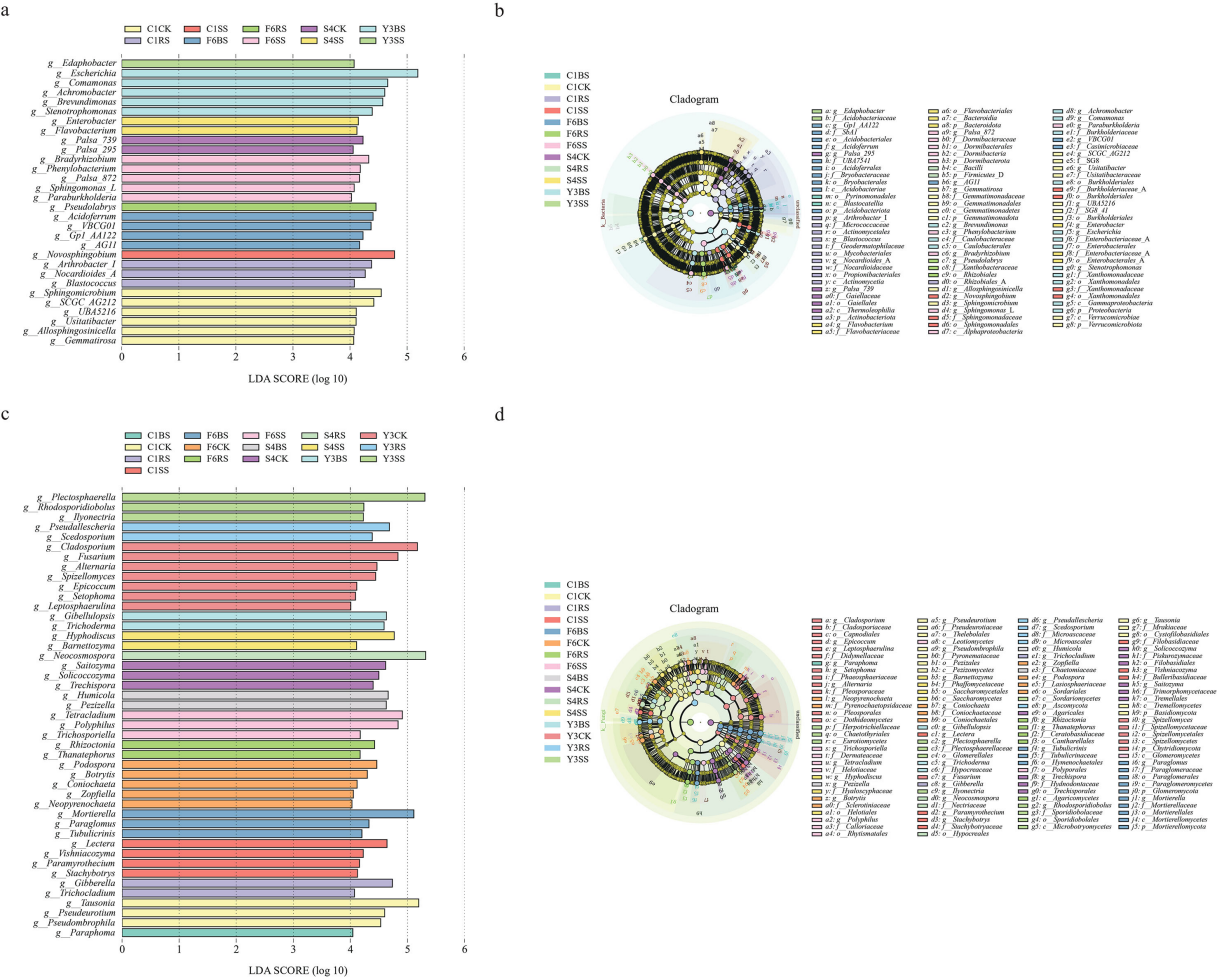
LEfSe analysis of soil samples from different compartments of medicinal plants. **(a, c)** LDA bar plots showing bacterial **(a)** and fungal **(C)** biomarkers. Bar length corresponds to LDA score (LDA > 4, p < 0.05). Colors indicate the specific species-compartment group in which each taxon is enriched, with groups comprising four plant species (F6: P *ginseng*; S4: P *quinquefolius*; Y3: P *notoginseng*; C1: A *bidentata*) and four soil compartments (CK, uncultivated control; BS, bulk soil; RS, rhizosphere soil; SS, rhizoplane soil). **(b, d)** Cladograms illustrating bacterial **(b)** and fungal **(d)** taxonomic relationships. Circles from center to periphery represent taxonomic levels from domain to genus. Colored nodes indicate taxa significantly enriched in specific species-compartment groups (colors correspond to groups in a, c); yellow nodes represent non-significant taxa. Colored background sectors highlight the taxonomic ranges of biomarkers for each group.

Analysis of the microbial baseline in uncultivated soils (**Figures 6A, C**) revealed that uncultivated soil for *Achyranthes bidentata* (C1CK) was characterized by bacterial genera such as *Allosphingosinicella* and *Sphingomicrobium.* Uncultivated soil for *Panax quinquefolius* (S4CK) was enriched with several uncultured candidate bacterial taxa (e.g., *Palsa-295* and *Palsa-739*). In contrast, uncultivated soil for *Panax notoginseng* (Y3CK) was rich in fungal taxa such as *Cladosporium* (LDA = 5.2) and *Fusarium* (LDA = 4.8).

Following cultivation, the microbial biomarkers in the plant root zones exhibited significant species-specific differentiation (**Figure 6B**). Analysis of bacterial biomarkers revealed that the rhizosphere of *Achyranthes bidentata* (C1RS) was predominantly characterized by *Actinobacteria*, including taxa with organic matter degradation potential such as *Nocardioides* (LDA = 4.3) and *Arthrobacter* (LDA = 4.4). In contrast, the characteristic taxa of *Panax* species were mainly concentrated in *Proteobacteria* and *Acidobacteriota*, such as *Pseudolabrys* (LDA = 4.5) in the rhizosphere of *Panax ginseng* (F6RS) and nitrogen-fixing related taxa like *Bradyrhizobium* (LDA = 4.3) in the rhizoplane soil (F6SS).

Analysis of fungal biomarkers further revealed species-specific differentiation patterns (**Figure 6D**). The rhizosphere of *Panax* species was significantly enriched with characteristic taxa associated with plant pathogens, primarily distributed within the *Nectriaceae* family of *Ascomycota*, including *Plectosphaerella* (LDA = 5.3) in the rhizosphere of *Panax notoginseng* (Y3RS), *Ilyonectria* (LDA = 4.2) in Y3SS, and *Neocosmospora* (LDA = 5.3) in the rhizosphere of *Panax quinquefolius* (S4RS). In contrast, the rhizosphere of *Achyranthes bidentata* (C1RS) was characterized by biocontrol and growth-promoting taxa, mainly within the *Hypocreales* order, including beneficial fungi such as *Vishniacozyma* (LDA = 4.2).

### 3.6 Identification of environmental drivers of microbial community structure

**Figure 7:**
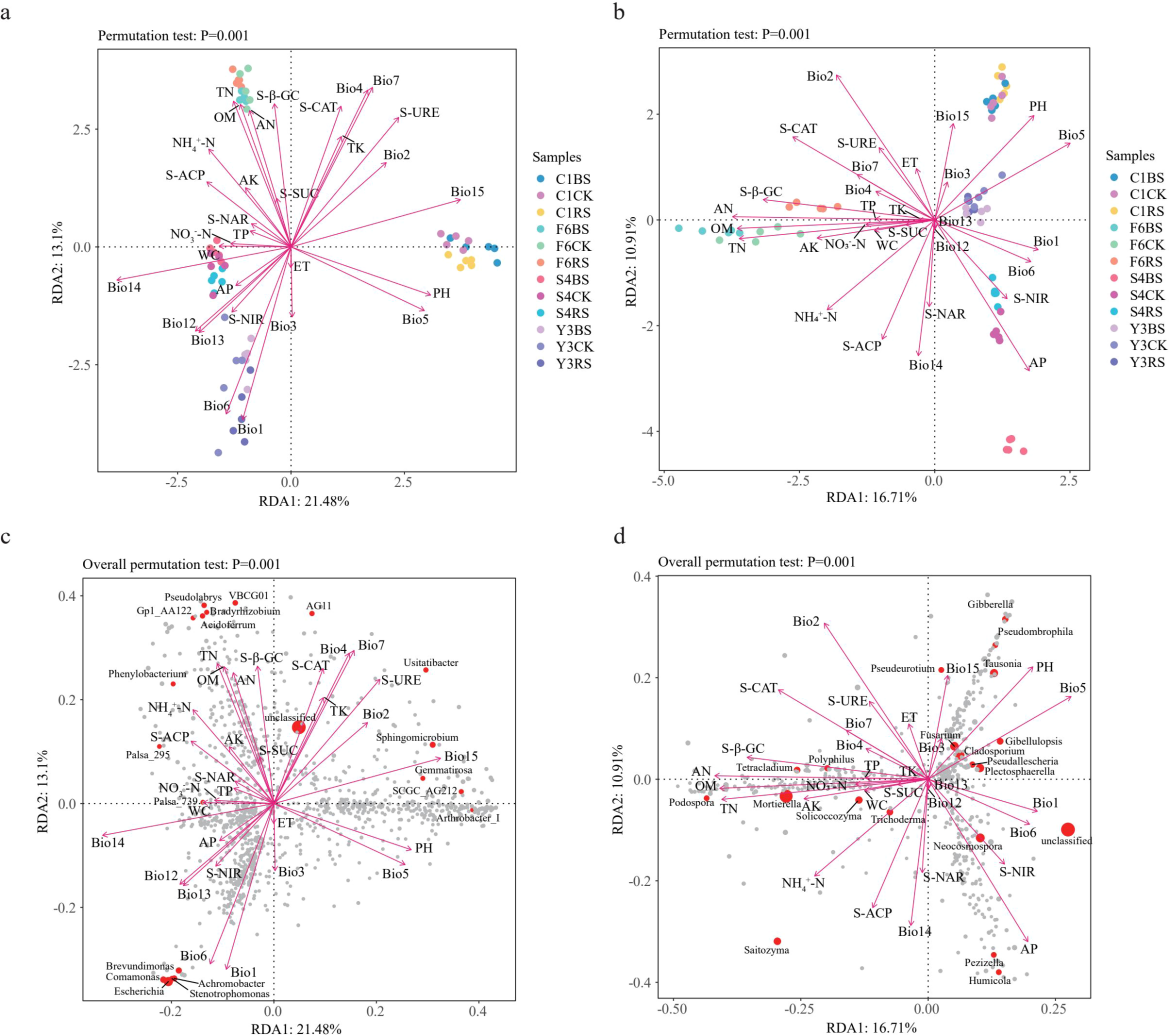
Redundancy analysis (RDA) of soil physicochemical properties and microbial communities. **(a, b)** RDA showing the effects of soil physicochemical properties on bacterial **(a)** and fungal **(b)** community structure. Colored points represent individual samples, with colors indicating the 16 species-compartment groups: four plant species (F6: P *ginseng*; S4: P *quinquefolius*; Y3: P *notoginseng*; C1: A *bidentata*) × four soil compartments (CK, uncultivated control; BS, bulk soil; RS, rhizosphere soil; SS, rhizoplane soil). Arrows represent soil physicochemical variables; arrow length indicates the strength of explanatory power, and arrow direction indicates the correlation with RDA axes. The angle between arrows reflects correlations among variables. Permutation tests confirmed model significance (p = 0.001), with explained variation of 34.5% for bacteria and 27.6% for fungi. **(d, d)** Correlation showing relationships between soil physicochemical properties and microbial genera for bacteria **(c)** and fungi **(d)**. Red points represent the top 20 most abundant genera, with point size proportional to relative abundance. Arrows represent soil physicochemical variables as in **(a, b)**. Sample points are colored by species-compartment groups as in **(a, b)**.

Having established plant species identity as the key determinant of microbial community structure, we employed redundancy analysis (RDA) to identify the specific environmental mechanisms underlying this differentiation. Permutation tests showed that the explanatory models of environmental factors for both bacterial and fungal communities reached extremely significant levels (P = 0.001), explaining 34.5% and 27.57% of the total variation, respectively (**Figure 7**).

The RDA results demonstrated that the microbial communities of the two plant types were driven by distinct environmental factors (**Figure 7A**). The bacterial community of *A. bidentata* showed a strong correlation with soil pH, with its samples clustering predominantly along the positive direction of the RDA1 axis. In contrast, *Panax* species exhibited more complex environmental driving patterns: the community structure of *P. ginseng* was closely associated with total nitrogen (TN) and organic matter (OM) content; *P. quinquefolius* communities were influenced by available phosphorus (AP) and nitrate nitrogen (NO_3_^-^-N); whereas *P. notoginseng* communities were primarily correlated with climatic factors, including annual mean temperature (Bio1) and minimum temperature of the coldest month (Bio6).

The fungal communities exhibited greater sensitivity to soil nitrogen and organic matter content (**Figure 7B**). The key drivers of fungal community differentiation were TAN (NH_4_^+^-N), OM, and TN, which demonstrated exceptionally high explanatory power (r² > 0.93). Specifically, the fungal community of *P. ginseng* showed positive correlations with all the aforementioned nitrogen and organic matter factors. In contrast, *P. quinquefolius* communities were associated with AP and S-NIR (abundance of the nitrite reductase gene). *P. notoginseng* communities were influenced by Bio5, while the root zone fungal community of *A. bidentata* was again confirmed to be primarily governed by pH.

Analysis at the genus level further validated the aforementioned environmental driving patterns (**Figures 7C, D**). Consistent with the soil nutrient-driven pattern, characteristic bacteria (*Pseudolabrys*, *Bradyrhizobium*) and fungi (*Mortierella*) in *P. ginseng* cultivated soils showed positive correlations with TN and OM. Taxa significantly enriched in the root zone of *P. notoginseng*, including *Stenotrophomonas*, *Achromobacter*, and *Plectosphaerella*, were positively correlated with temperature factors (Bio5, Bio6). The characteristic fungus *Neocosmospora* in the rhizosphere of *P. quinquefolius* (S4RS) exhibited a significant positive correlation with AP. In contrast, characteristic bacteria (*Arthrobacter*) and fungi (*Gibberella*) in *A. bidentata* cultivated soils were closely associated with pH.

### 3.7 Microbial network structure and root zone microecological stability

**Figure 8:**
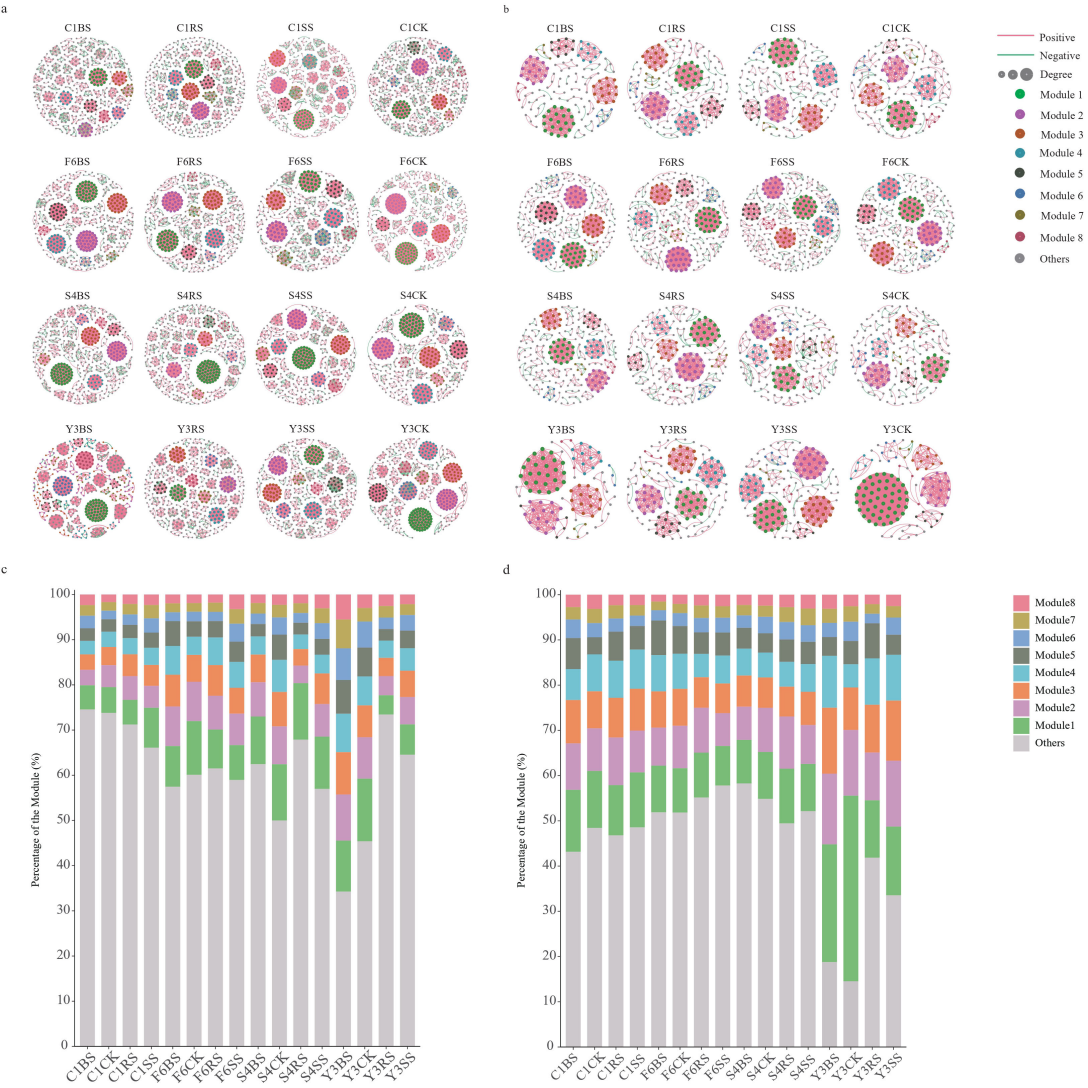
Co-occurrence network analysis of microbial communities in different soil compartments of medicinal plants. **(a, b)** Co-occurrence networks for **(a)** bacterial and **(b)** fungal communities. Only robust (|r| > 0.8) and statistically significant (*p* < 0.05) correlations are shown. Node size represents connectivity degree; edge color indicates positive (red) and negative (green) correlations, with thickness proportional to the correlation coefficient. Modules 1–8 are labeled in descending order of node abundance, with remaining modules shown in gray. **(c, d)** Module distribution proportions showing the relative abundance of each module in bacterial **(c)** and fungal **(d)** co-occurrence networks, presented as percentages. Colors correspond to modules identified in **(a, b)**.

To decipher the interaction patterns among microorganisms in the root zones of different medicinal plants, this study constructed microbial co-occurrence networks based on all ASVs (|r| > 0.8, p < 0.05). Comparative analysis of the network structural characteristics between *Panax* species and *Achyranthes bidentata* revealed significant differences in network complexity, stability, and modular structure (**Figure 8**).

**Figure 9:**
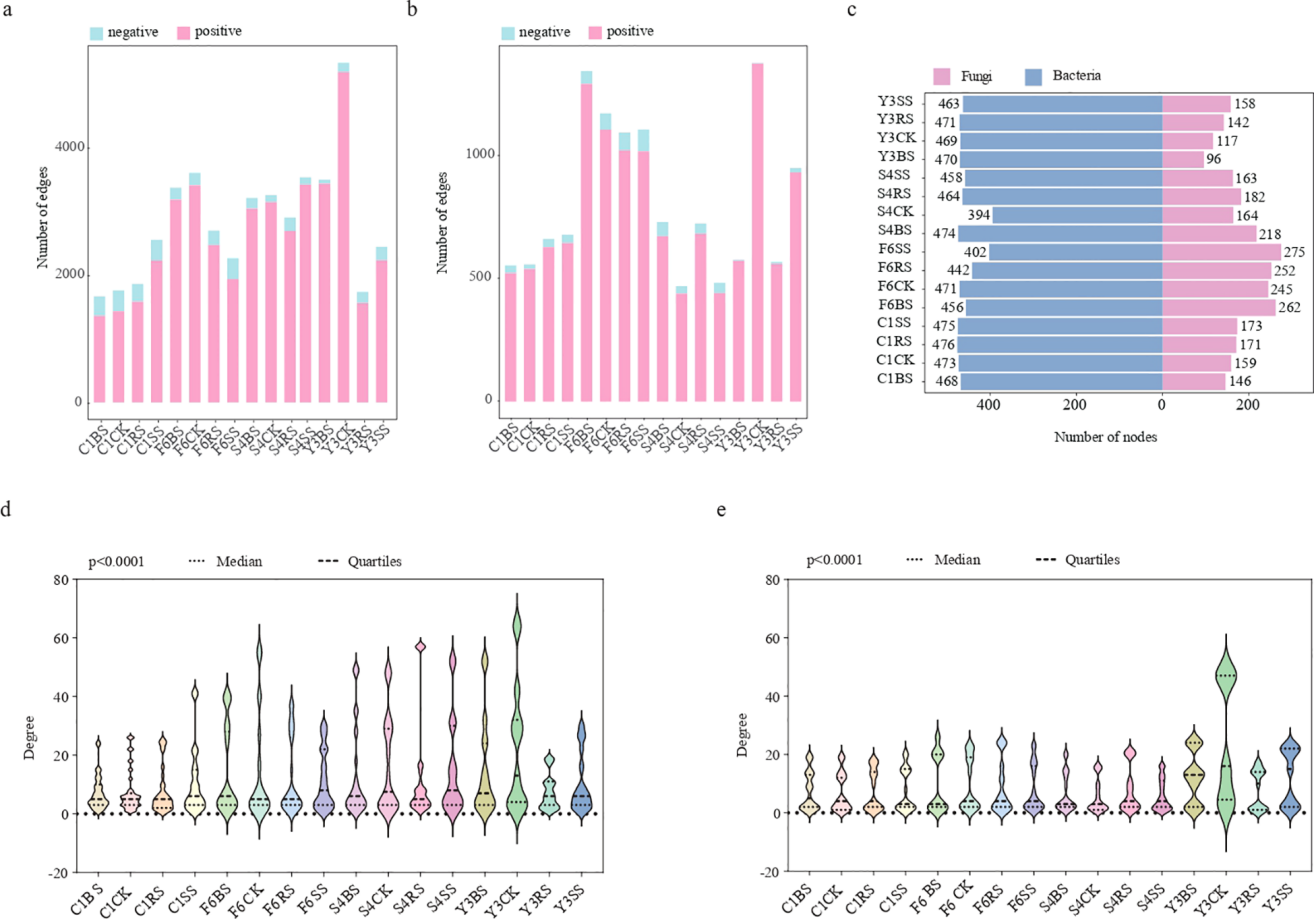
Network properties of co-occurrence networks for different soil compartments of medicinal plants. **(a, b)** Stacked bar plots showing the number of edges in bacterial **(a)** and fungal **(b)** co-occurrence networks. Red and green segments represent positive and negative correlations, respectively. Each bar represents one species-compartment group: four plant species (F6: P *ginseng*; S4: P *quinquefolius*; Y3: P *notoginseng*; C1: A *bidentata*) × four soil compartments (CK, uncultivated control; BS, bulk soil; RS, rhizosphere soil; SS, rhizoplane soil). **(c)** Number of nodes in bacterial and fungal co-occurrence networks across all species-compartment groups. **(d, e)** Boxplots showing degree distribution differences among groups for bacterial **(d)** and fungal **(e)** communities. Different colors represent different plant species as in **(a, b)**. Significance was determined by non-parametric Wilcoxon tests (p<0.0001).

Analysis of bacterial network structure revealed that the root zone bacterial network of *Achyranthes bidentata* remained relatively stable after cultivation, showing no significant difference in node number compared to uncultivated soil, with limited increase in connectivity (5.8%-45.2%) and maintaining a high proportion of negative connections (12.7%-18.6%). In contrast, the root zone bacterial networks of *Panax* species underwent significant remodeling: the connectivity in the rhizosphere of *Panax notoginseng* (Y3RS) decreased sharply by 67.4%, and the proportion of negative connections in the rhizoplane soil of *Panax quinquefolius* (S4SS) dropped significantly to 3.2% (**Figures 9A, C**). Topological analysis (**Supplementary Table 8**) indicated that the bacterial networks in the root zones of *Panax* species exhibited higher connectivity density (0.016-0.034) and average degree (7.363-15.419), which showed a decreasing trend relative to uncultivated soil. In comparison, the root zones of *A. bidentata* maintained relatively sparse connectivity density (0.015-0.023) and lower average degree (7.111-10.741).

The fungal network structure exhibited similar species-specific patterns (**Figures 9b, c**). The fungal network metrics in the root zone of *Achyranthes bidentata* remained stable, whereas significant changes were observed in *Panax* species: the node number in cultivated soils of *Panax* species generally showed an increasing trend, while connectivity in *Panax notoginseng* decreased by 31.1%-58.5%. However, both connectivity density (0.057-0.127) and average degree (8.014-12.051) were significantly higher than those in *A. bidentata* (connectivity density: 0.045-0.052; average degree: 7.031-7.861), reflecting tighter microbial interactions in *Panax* species (**Supplementary Table 8**). We further evaluated the degree distribution ranges to identify hub nodes (**Figures 9D, E**). Bacterial and fungal networks of *Achyranthes bidentata* showed narrow degree ranges with uniform structure, whereas Panax species exhibited wider distributions, with some samples harboring highly connected hubs.

Modularity analysis further revealed differences in network stability (**Supplementary Table 9**). The root zone microbial networks of *Panax* species showed significantly enhanced modularity: the bacterial modularity index increased by 20.3% and the fungal modularity index by 154.2% in Y3RS, while the bacterial modularity index increased by 18.7% in F6SS. Concurrently, the increased Gini coefficient and decreased evenness of module distribution indicated enhanced heterogeneity in the modular structure. In contrast, neither the modularity indices nor the degree of module variation changed significantly in the root zone microbial networks of *A. bidentata*, demonstrating maintained structural stability.

Analysis of keystone species interaction patterns corroborated the structural differences observed in the networks (**Supplementary Figure 4**). In bacterial networks, high-abundance taxa exhibited species-specific connection patterns. For instance, *Sphingomicrobium* primarily formed positive connections in the root zone of *Achyranthes bidentata*, but displayed more negative connections in that of *Panax ginseng* (**Supplementary Figure 4A**). Fungal core taxa demonstrated even more distinct niche-specific interaction patterns, as exemplified by *Fusarium*, whose connection patterns showed a continuum from entirely positive to entirely negative correlations across different samples. Notably, the biomarkers *Ilyonectria* and *Neocosmospora* in sample Y3SS showed a significant negative correlation, suggesting ecological niche competition between these two pathogenic fungi (**Supplementary Figure 4B**).

### 3.8 Divergent metabolic potential in the root zone revealed by microbial function prediction

**Figure 10:**
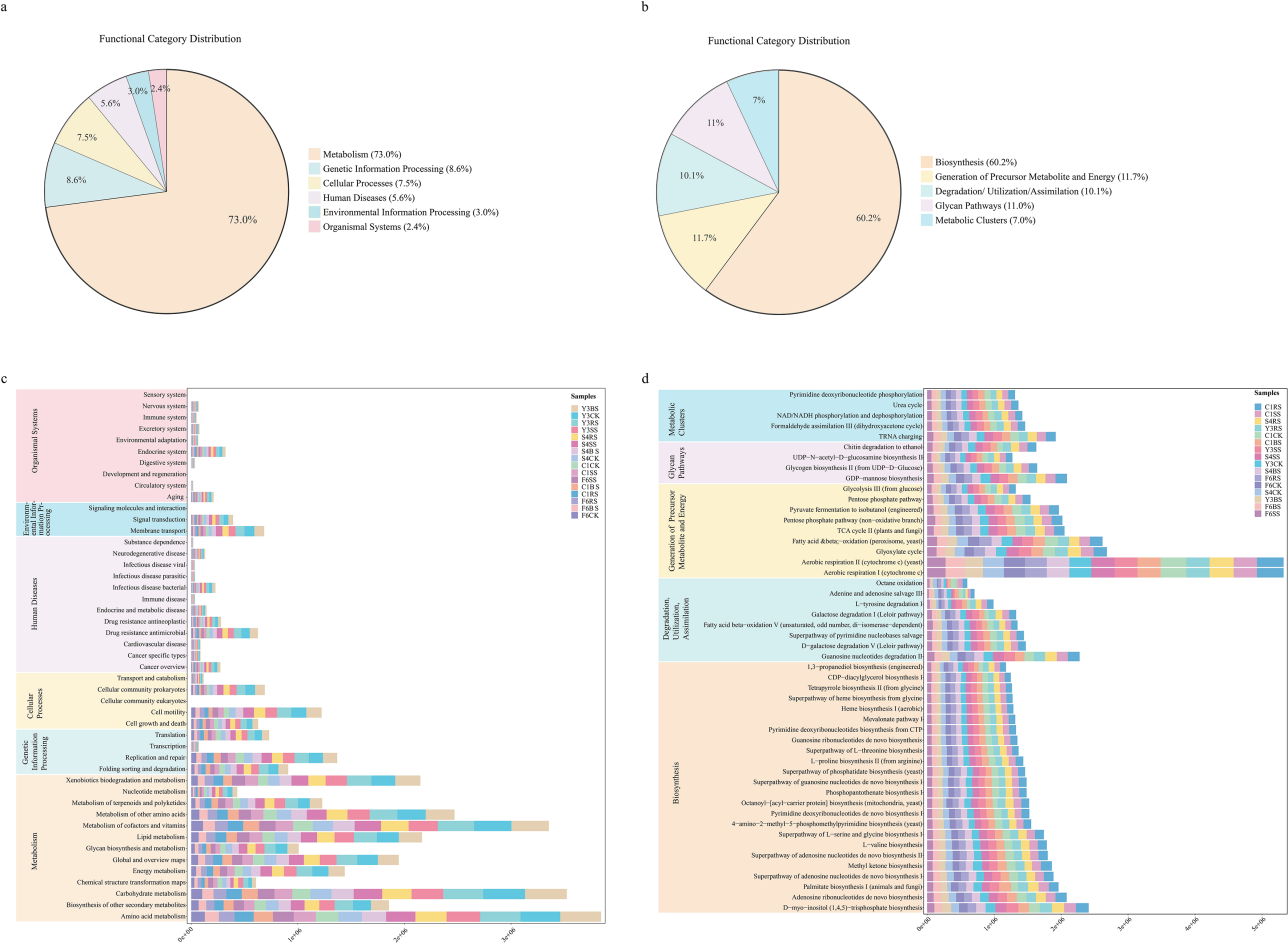
Microbial function prediction in different soil compartments of medicinal plants. **(a)** Relative abundance of KEGG level 1 functional pathways in bacterial communities predicted by PICRUSt2. Different colors represent different level 1 pathway categories as shown in the legend. **(b)** Relative abundance of MetaCyc major functional pathways in fungal communities. Different colors represent different level 1 pathway categories as shown in the legend. **(c, d)** Horizontal stacked bar plots showing the relative abundance of KEGG level 2 functional pathways in bacterial (c) and MetaCyc secondary metabolic pathways in fungal (d) communities. The y-axis lists individual pathways; the x-axis represents the total relative abundance summed across samples, with different colors within the bar representing different species-compartment groups: four plant species (F6: P *ginseng*; S4: P *quinquefolius*; Y3: P *notoginseng*; C1: A *bidentata*) × four soil compartments (CK, uncultivated control; BS, bulk soil; RS, rhizosphere soil; SS, rhizoplane soil). Bar length indicates the total abundance of each pathway across all groups, and colored segments show the contribution of each species-compartment group to that pathway.

To assess the impact of medicinal plant cultivation on root zone microbial function, this study conducted functional prediction of bacterial and fungal communities using PICRUSt2 and MetaCyc databases (**Figure 10**).

Bacterial functional prediction revealed that Metabolism dominated the Kyoto Encyclopedia of Genes and Genomes (KEGG) level 1 categories (73.0 %), followed by Genetic Information Processing (8.6 %) and Cellular Processes (7.5 %) (**Figure 10A**). Compared to C1CK, the functional profile of *A. bidentata* root zones showed an overall decreasing trend, with abundance in Metabolism decreasing by 17.4 % in C1RS and 15.0 % in C1BS. In contrast, *Panax* species exhibited species−specific functional differentiation: Metabolism abundance increased significantly by 54.2 % in F6SS and by 34.8 % in S4RS, while cultivated soils of P. notoginseng showed generally decreased functional abundance (**Figure S3 A–F**).

At the secondary metabolism level, changes in Metabolism of terpenoids and polyketides were particularly prominent (**Figure 10C**). *Panax* species showed widespread enhancement of this function in root zones, with F6SS increasing by 74.8%, S4RS soil rising by 29.5%, and Y3RS soil increasing by 7.8%. In contrast, *A. bidentata* root zones exhibited an overall inhibitory trend, with C1RS and C1BS decreasing by 10.5% and 9.0%, respectively.

Fungal functional analysis revealed that among the five major metabolic pathways based on the MetaCyc database, Biosynthesis accounted for the highest proportion (60.2%) (**Figure 10B**). The root zone of *A. bidentata* maintained a relative advantage in Degradation/Utilization/Assimilation and Metabolic Clusters pathways, with the abundance of Metabolic Clusters pathways in C1RS increasing by 8.8% compared to C1CK. In contrast, *Panax* species exhibited distinct metabolic characteristics: S4RS showed a 28.3% increase in Biosynthesis pathways, while Y3RS increased by 13.4% in Generation of Precursor Metabolite and Energy pathways (**Supplementary Figures 3G**-**K**).

Notably, the rhizosphere soils of all four medicinal plants exhibited higher abundance trends across the five level 1 functional pathways compared to other soil compartments. Particularly in Energy metabolism, the abundance of Aerobic respiration I (cytochrome c) and Aerobic respiration II (cytochrome c) (yeast) in the rhizosphere soils of *Panax* species increased by an average of 18.6% relative to uncultivated soil, while changes in the root zone of *A. bidentata* were relatively moderate (**Figure 10D**).

## 4 Discussion

This study provides a multi-dimensional analysis of rhizosphere microecology across medicinal plants with contrasting susceptibilities to continuous cropping. By integrating soil chemistry, microbial community structure, interaction networks, and functional potential, our findings suggest that *Panax* species and *Achyranthes bidentata* may adhere to fundamentally divergent ecological strategies. Below, we synthesize these findings into a coherent framework, arguing that the resource-acquisitive strategy of *Panax* species, while optimizing short-term nutrient capture, renders their rhizosphere micro-ecosystem inherently unstable and prone to CCP. Conversely, the resource-conservative strategy of A. *bidentata* fosters a resilient and stable system capable of sustaining long-term monoculture.

### 4.1 Species-specific soil microenvironment modification: the chemical basis of ecological strategies

Our paired-site comparative analysis using Log_2_ fold change between cultivated and uncultivated soils revealed that *Panax* species systematically remodeled root zone chemistry. All three *Panax* species significantly decreased soil pH and promoted ammonium nitrogen accumulation (e.g., Log_2_ FC = +1.64 in P. *notoginseng* rhizosphere, Y3RS), while inhibiting nitrate reductase and nitrite reductase activities. This acidification is consistent with previous reports of organic acid exudation (e.g., citric, malic, phenolic acids) by *Panax* roots (Chen et al., 2023; Li et al., 2024), which selectively enrich acid-tolerant bacteria such as *Acidobacteria* and disrupt nitrification. While the observed soil chemical changes strongly implicate root exudate-mediated processes, direct characterization of exudate compounds was beyond the scope of the current study. Future investigations integrating metabolomic analysis of root exudates with metagenomic sequencing will be necessary to establish causal links between specific exudate compounds and the microbial assembly patterns reported here. The concurrent reduction in total and available phosphorus (e.g., Log_2_FC = –1.88 for AP in Y3RS) suggests enhanced phosphorus uptake or immobilization, further reflecting an aggressive nutrient-acquisitive strategy.

In contrast, A. *bidentata* exhibited minimal chemical perturbation, maintaining neutral pH while specifically enhancing available phosphorus (Log_2_ FC = +1.74 in bulk soil, C1BS) and nitrate nitrogen (Log_2_ FC = +0.74 in rhizosphere, C1RS). This targeted regulation aligns with the neutral to weakly alkaline nature of its root exudates (e.g., polysaccharides, alkaloids) (Wang, 2020) and the enrichment of *Actinobacteria* known for phosphate solubilization without acidification (Sabaté and Brandán, 2022). The conservative strategy thus achieves efficient nutrient mobilization while preserving baseline soil properties.

### 4.2 Species-specific microbial community assembly: biological manifestation of ecological strategies

The chemical modifications imposed by each plant act as strong environmental filters, resulting in distinct microbial assemblies. *Panax* species consistently assembled fungal-dominated communities, with increased fungal ASV counts (net +576 in P. *ginseng*) and decreased bacterial ASV numbers (up to –39.0% in rhizoplane). This pattern likely stems from antimicrobial root exudates (e.g., ginsenosides) that suppress bacteria (Yang et al., 2022). However, LEfSe analysis revealed significant enrichment of pathogenic Nectriaceae (*Ilyonectria*, *Neocosmospora*, *Plectosphaerella*; LDA > 4.2), known to cause root rot in *Panax* (Sun et al., 2023), —a critical trade-off of the acquisitive strategy.

By contrast, A. *bidentata* fostered bacterial-dominated communities with significant increases in bacterial ASV counts (+57.4% in bulk soil, C1BS) and enriched beneficial taxa such as *Actinobacteria* (*Nocardioides*, *Arthrobacter*; LDA > 4.3) and Hypocreales fungi (*Vishniacozyma*; LDA = 4.2). These taxa possess dual functions in organic matter degradation and pathogen suppression (Chen et al., 2020; Hong et al., 2015), providing an integrated “nutrient activation–biocontrol” service that underpins the conservative strategy’s resilience. PERMANOVA confirmed that plant species identity explained the majority of community variation (bacteria: 60.5%, fungi: 46.2%), overwhelmingly exceeding soil compartment effects, underscoring the dominant role of plant strategy in microbiome assembly.

### 4.3 Ecological strategy divergence at the system level: network stability and functional potential

Microbial co-occurrence networks captured system-level consequences of the two strategies. Although these networks represent statistical associations rather than direct ecological interactions, shifts in their topology can signal changes in community assembly rules and potential stability. In A. *bidentata*, network structure remained stable after cultivation, with negative correlation proportions ranging from 12.7% to 18.6%, indicating preserved competitive interactions that limit pathogen spread and enhance functional redundancy. This “high modularity–low connectivity” topology is characteristic of disturbance-resistant ecosystems (Zhang et al., 2018) and aligns with the conservative strategy’s emphasis on long-term stability.

In contrast, *Panax* species exhibited profound network restructuring, characterized by reduced negative correlations (as low as 3.2% in P. *quinquefolius* rhizoplane, S4SS) despite higher connectivity density. The sharp decline in negative edges suggests disruption of competitive exclusion, potentially facilitating pathogen establishment. Concurrently, modularity increased (e.g., +20.3% in P. *notoginseng* rhizosphere, Y3RS), reflecting intensified niche differentiation driven by strong environmental filtering (Sun et al., 2023). This unstable network configuration, coupled with pathogen enrichment, creates a feedback loop that undermines ecosystem resilience under continuous cropping.

Functional prediction further highlighted strategic divergence. *Panax* species markedly activated secondary metabolic pathways, particularly terpenoid and polyketide metabolism (up to +74.8% in P. ginseng rhizoplane). This specialization reflects co-evolutionary adaptation supporting host secondary metabolite synthesis (Yu et al., 2024) but reduces functional redundancy, increasing vulnerability to perturbation. Conversely, A. *bidentata* maintained conserved metabolic profiles, with relative advantages in degradation and utilization pathways—key features of a conservative strategy (Ramond et al., 2024).

### 4.4 Integrating mechanisms: a predictive framework for CCP susceptibility

**Figure 11:**
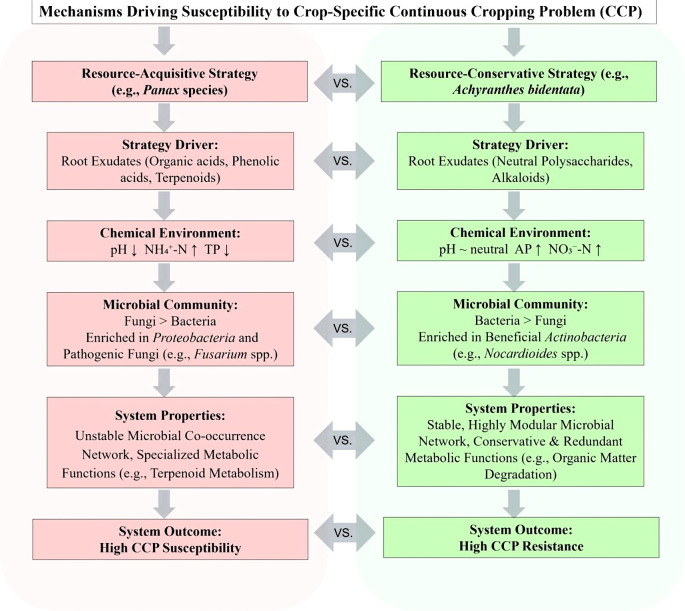
Conceptual model of divergent rhizosphere ecological strategies and their consequences for continuous cropping problem (CCP) susceptibility. The model contrasts two contrasting strategies: the resource-acquisitive strategy (left panel) employed by CCP-sensitive Panax species (P. *ginseng*, P. *quinquefolius*, P. *notoginseng*) and the resource-conservative strategy (right panel) employed by CCP-resilient *Achyranthes bidentata*. Key features of the acquisitive strategy include soil acidification, ammonium accumulation, fungal-dominated microbiomes, enrichment of pathogenic Nectriaceae, unstable co-occurrence networks with low negative correlations, and activated secondary metabolism. Key features of the conservative strategy include neutral pH maintenance, nitrate and available phosphorus enrichment, bacterial-dominated microbiomes with beneficial Actinobacteria, stable co-occurrence networks with moderate negative correlations, and conserved metabolic profiles. Arrows indicate proposed causal pathways linking plant ecological strategies to rhizosphere microbiome assembly and ultimately to CCP susceptibility or resilience. CCP, continuous cropping problem.

Synthesizing these findings, we propose a mechanistic framework linking plant ecological strategy to CCP susceptibility (**Figure 11**). The data suggest that the putative acquisitive strategy of *Panax* species is associated with rhizosphere acidification and ammonium accumulation, creating selective pressures that assemble fungal-dominated microbiomes enriched in pathogens. These communities form unstable networks with reduced competitive interactions and adopt metabolic profiles prioritizing secondary metabolism. While maximizing short-term nutrient acquisition, this configuration’s low functional redundancy, network instability, and pathogen load render it highly susceptible to monoculture stresses.

In contrast, the conservative strategy of A. *bidentata* maintains abiotic stability through neutral pH and targeted nutrient enhancement, favoring diverse bacterial-dominated communities rich in beneficial *Actinobacteria* with dual nutrient–pathogen functions. These communities assemble into stable, modular networks with preserved competitive interactions and retain broad catabolic functions. This configuration prioritizes functional redundancy, interaction robustness, and systemic resilience, thereby buffering against continuous cropping disturbances and explaining its low CCP susceptibility.

This framework shifts the understanding of CCP from a descriptive, pathogen-centric view to a predictive, strategy-based theory. It provides a scientific basis for developing tailored soil management practices—such as precision microbiome engineering or crop rotation design—aligned with the inherent ecological strategy of the target medicinal plant, paving the way for sustainable cultivation.

### 4.5 Study limitations and future perspectives

This study has certain limitations. Although a paired sampling design (cultivated soil vs. adjacent uncultivated control soil) was employed, which effectively eliminates background differences inherent to the sampling sites themselves to isolate plant-induced changes, it must be acknowledged that each medicinal plant species was sourced from its respective primary, yet geographically separate, cultivation area. Consequently, plant species and geographic environmental factors (e.g., climate, soil parent material) are confounded to some extent. While climatic factors were included in the analysis, and the three *Panax* species, originating from different climatic zones, exhibited similar trends—providing some support for the dominant role of plant species—the potential influence of regional environmental background on the observed interspecific differences cannot be entirely ruled out. Future research should compare different species under identical environmental conditions (e.g., within a common garden) or analyze multiple geographic populations of the same species to more precisely disentangle the relative contributions of plant genetics and regional environment to rhizosphere microbiome assembly.

Additionally, the functional profiles presented in this study are predictions inferred from marker gene data (PICRUSt2 for bacteria; MetaCyc for fungi) and should be considered exploratory. They provide hypotheses about potential microbial functions but require validation using metagenomic, metatranscriptomic, or targeted metabolomic approaches to confirm actual gene expression and metabolic activity.

## 5 Conclusion

This study suggests that the contrasting susceptibilities may originate from a fundamental divergence in rhizosphere ecological strategies. *Panax* species adopt a resource-acquisitive strategy, characterized by aggressive soil acidification and ammonium accumulation alongside a concurrent inhibition of nitrification enzymes. This process drives the assembly of specialized, fungal-dominated microbiomes enriched with pathogenic Nectriaceae taxa. These communities form metabolically active but unstable interaction networks, characterized by significantly reduced competitive interactions and an activated secondary metabolism; this renders the ecosystem inherently vulnerable to pathogen enrichment and functional decline under monoculture. In contrast, *Achyranthes bidentata* employs a resource-conservative strategy that maintains a neutral pH while specifically enhancing available phosphorus and nitrate nitrogen. This environment fosters bacterial-dominated communities enriched with beneficial *Actinobacteria* and Hypocreales fungi. These communities assemble into stable, modular networks that preserve competitive interactions and retain broad catabolic functions, which confers high systemic resilience and continuous cropping tolerance. Plant species identity was a far stronger determinant of microbial community structure than soil compartment effects, confirming that host ecological strategy is the primary driver of rhizosphere assembly.

Future research should directly characterize root exudate profiles to establish causal links between specific compounds and the observed microbial assembly patterns. Long-term monoculture experiments are also needed to track the temporal dynamics of network destabilization and pathogen enrichment. Functional validation of predicted metabolic pathways through metagenomic and metatranscriptomic approaches would further strengthen the proposed mechanistic framework. Extending this comparative strategy to additional medicinal plant species with varying susceptibilities to continuous cropping will test its predictive power and facilitate the development of tailored microbiome management practices for sustainable cultivation.

## Data Availability

The datasets presented in this study can be found in online repositories. The names of the repository/repositories and accession number(s) can be found below: https://www.ncbi.nlm.nih.gov/, https://www.ncbi.nlm.nih.gov/bioproject/PRJNA1404892 https://www.ncbi.nlm.nih.gov/, https://www.ncbi.nlm.nih.gov/bioproject/PRJNA1405116.
