# Supplementary material for: Ecological strategies determine continuous cropping susceptibility in Panax and Achyranthes
Source: Front Plant Sci. 2026 Apr 14;17:1791596. doi: 10.3389/fpls.2026.1791596 (PMC13121161; doi:10.3389/fpls.2026.1791596)
Supplement: Supplementary file 1 [file DataSheet1.docx]

**Supplementary_Material**

**Table S1**. Detailed information of plant samples and sampling sites

| Plant Species | Cult. Year | Location | Altitude | Soil Type | Sampling Time | Coordinates |
| --- | --- | --- | --- | --- | --- | --- |
| P. *ginseng* | 6 | Fusong, JL | 768 m | Phaeozem | Sep. 2023 | 42.18°N, 127.55°E |
| P. *quinquefolius* | 4 | Rongcheng, SD | 40 m | Arenosol | Sep. 2022 | 36.93°N, 122.26°E |
| P. *notoginseng* | 3 | Wenshan, YN | 1374 m | Ferralosol | Nov. 2022 | 23.34°N, 104.24°E |
| A. *bidentata* | 1 | Harqin Banner, IM | 630 m | Sandy Loam | Oct. 2023 | 42.15°N, 118.81°E |

**Note**: Cult. Year, cultivation year; Coordinates, latitude and longitude; Location abbreviations: JL (Jilin province), SD (Shandong province), YN (Yunnan province), IM (Inner Mongolia Autonomous Region); Plant species are abbreviated according to standard botanical nomenclature.

**Table S2**. Soil sample naming conventions.

| Sample Source | soil compartment | Abbreviation |
| --- | --- | --- |
| P. *ginseng* （F6） | Rhizoplane Soil | F6SS |
|  | Rhizosphere Soil | F6RS |
|  | Bulk Soil | F6BS |
|  | Uncultivated Control Soil | F6CK |
| P. *quinquefolius* (S4) | Rhizoplane Soil | S4SS |
|  | Rhizosphere Soil | S4RS |
|  | Bulk Soil | S4BS |
|  | Uncultivated Control Soil | S4CK |
| P. *notoginseng* (Y3) | Rhizoplane Soil | Y3SS |
|  | Rhizosphere Soil | Y3RS |
|  | Bulk Soil | Y3BS |
|  | Uncultivated Control Soil | Y3CK |
| A. *bidentata* (C1) | Rhizoplane Soil | C1SS |
|  | Rhizosphere Soil | C1RS |
|  | Bulk Soil | C1BS |
|  | Uncultivated Control Soil | C1CK |

**Note:** The sample code (e.g., F6) combines location abbreviation and cultivation years. The full sample abbreviation is formed by appending the soil niche code (SS, RS, BS, CK) to the sample code.

**Table S3**. Soil physicochemical properties of different medicinal plants from various ecological niches

| Sample | soil compartment | WC (%) | PH | OM (g/kg) | TN (g/kg) | TP (g/kg) | TK (g/kg) |
| --- | --- | --- | --- | --- | --- | --- | --- |
| C1 | RS | 8.482±0.585gh | 7.110±0.014b | 5.612±0.213ef | 0.490±0.005g | 0.750±0.040b-e | 23.608±1.122ab |
|  | BS | 9.340±0.052fh | 7.300±0.028a | 5.538±0.029ef | 0.500±0.006fg | 0.690±0.021e | 20.188±0.816abc |
|  | CK | 10.800±0.055eg | 7.258±0.046ab | 5.322±0.263ef | 0.482±0.008g | 0.860±0.041a-e | 18.460±0.233bc |
| S4 | RS | 13.018±0.766def | 6.138±0.005de | 8.680±0.158c | 0.808±0.013d | 0.808±0.010d | 19.730±0.641abc |
|  | BS | 13.972±0.187d | 6.210±0.038df | 5.092±0.140ef | 0.552±0.007e | 0.744±0.005e | 20.400±0.131a |
|  | CK | 15.262±0.555d | 6.402±0.013f | 7.110±0.098d | 0.822±0.005d | 0.842±0.015cd | 17.320±0.418c |
| Y3 | RS | 30.800±0.329ab | 6.150±0.005de | 6.185±0.0.315def | 0.552±0.011ef | 0.940±0.016abc | 7.500±0.122d |
|  | BS | 31.040±0.528ab | 6.158±0.010de | 4.190±0.505f | 0.472±0.007g | 0.824±0.005d | 7.542±0.250d |
|  | CK | 31.940±0.073a | 6.700±0.016c | 7.680±0.474cde | 0.600±0.018e | 0.990±0.008a | 7.100±0.316d |
| F6 | RS | 30.578±0.077b | 5.804±0.071eg | 33.420±0.158b | 2.430±0.014c | 0.958±0.015ab | 20.808±0.191a |
|  | BS | 27.398±0.074c | 5.758±0.029g | 35.710±0.063a | 2.570±0.008b | 0.900±0.026a-d | 20.980±0.313a |
|  | CK | 28.11±0.291c | 6.232±0.019d | 35.810±0.032a | 2.672±0.007a | 1.010±0.028a | 20.620±0.256a |

**Table S3** (continued). Soil physicochemical properties of different medicinal plants from various ecological niches

| Sample | Ecological niches | AN (mg/kg) | AP (mg/kg) | AK (mg/kg) | NO₃⁻-N (mg/kg) | NH₄⁺-N (mg/kg) |
| --- | --- | --- | --- | --- | --- | --- |
| C1 | RS | 34.300±0.221g | 6.138±0.316g | 115.852±0.926e | 2.640±0.358e | 1.882±0.064fg |
|  | BS | 41.300±0.111c | 13.240±0.284f | 114.320±0.464ef | 2.298±0.191e | 1.580±0.239fgh |
|  | CK | 35.468±0.639efg | 3.972±0.164hi | 95.480±0.597g | 1.582±0.038e | 1.708±0.039gh |
| S4 | RS | 38.968±0.639c-f | 160.258±3.070a | 106.639±1.598f | 6.882±0.582d | 28.268±0.192b |
|  | BS | 25.938±0.208h | 180.536±5.209a | 189.238±11.778cd | 4.640±0.667de | 15.230±0.268e |
|  | CK | 35.288±0.064fg | 98.396±1.488b | 58.338±1.541h | 5.248±0.386d | 18.140±0.291d |
| Y3 | RS | 34.300±0.553g | 21.338±0.568e | 190.622±4.293d | 26.948±0.123a | 3.308±0.465fgh |
|  | BS | 37.800±0.332de | 60.770±0.733d | 207.828±7.331cd | 25.068±0.864abc | 1.968±0.022f |
|  | CK | 41.300±0.664cd | 78.812±0.040c | 170.558±3.428d | 22.638±0.628bc | 1.058±0.119h |
| F6 | RS | 140.468±0.639b | 3.322±0.117ij | 281.808±5.336b | 22.568±0.158b | 18.488±1.534cde |
|  | BS | 164.968±2.785a | 2.742±0.185j | 216.200±3.033c | 20.622±0.173c | 34.238±0.106a |
|  | CK | 154.468±1.278a | 5.142±0.214gh | 360.922±2.839a | 26.168±0.347a | 20.548±0.086c |

**Note:** The robustness of differences in group means was tested using Welch's ANOVA. Data are presented as mean ± standard deviation (SD). Post-hoc multiple comparisons were performed using the Games-Howell method, which does not assume equal variances. Different superscript letters within the same row indicate significant differences among groups at p<0.05; groups sharing the same superscript letter are not significantly different. For simplicity, consecutive letter ranges (e.g., a-e, b-e) are used to represent extended strings of superscript letters.

**Table S4**. Soil enzyme activities of different medicinal plants from various ecological niches

| Sample | Ecological niches | S_β_GC (U/g) | S_CAT (U/g) | S_URE (U/g) | S_ACP (U/g) | S_NAR (U/g) | S_NIR (U/g) | S_SUC (U/g) |
| --- | --- | --- | --- | --- | --- | --- | --- | --- |
| C1 | RS | 9.362±0.727cd | 0.630±0.011c | 0.750±0.000b | 0.258±0.002f | 0.102±0.004efg | 35.708±1.714def | 1.100±0.000c |
|  | BS | 10.610±0.197c | 0.638±0.007c | 0.800±0.003a | 0.210±0.000h | 0.110±0.003ef | 32.972±0.108f | 1.080±0.009c |
|  | CK | 10.560±0.011c | 0.670±0.005c | 0.800±0.009ab | 0.168±0.002i | 0.090±0.005fgh | 50.302±2.246bc | 0.942±0.005d |
| S4 | RS | 6.372±0.565de | 0.288±0.007d | 0.362±0.008e | 1.168±0.017b | 0.240±0.003c | 60.808±3.097b | 0.268±0.014fg |
|  | BS | 4.424±0.521ef | 0.108±0.007g | 0.248±0.002f | 1.000±0.032bcd | 0.310±0.030bcd | 53.978±1.052b | 0.332±0.002f |
|  | CK | 10.930±0.183c | 0.108±0.011g | 0.258±0.008f | 1.562±0.027a | 0.608±0.049a | 79.238±0.492a | 0.580±0.003e |
| Y3 | RS | 6.470±0.994c-f | 0.200±0.003ef | 0.102±0.005h | 0.360±0.006e | 0.068±0.007gh | 54.644±2.396b | 0.230±0.006g |
|  | BS | 3.286±0.237f | 0.210±0.005e | 0.090±0.000h | 0.222±0.002g | 0.078±0.004h | 56.072±2.060b | 0.180±0.000h |
|  | CK | 6.200±0.041de | 0.174±0.005f | 0.140±0.003g | 0.180±0.003i | 0.120±0.003e | 52.960±0.781b | 0.160±0.003i |
| F6 | RS | 30.030±0.703a | 1.050±0.014a | 0.870±0.014a | 0.972±0.016cd | 0.192±0.004d | 35.418±0.339e | 2.688±0.008a |
|  | BS | 29.392±0.650a | 0.948±0.002b | 0.716±0.005c | 1.006±0.015c | 0.210±0.008cd | 36.752±0.432de | 2.192±0.036b |
|  | CK | 22.380±0.508b | 0.928±0.004b | 0.572±0.002d | 0.920±0.008d | 0.270±0.005b | 39.718±0.581cd | 2.030±0.075b |

**Note**: The robustness of differences in group means was tested using Welch's ANOVA. Data are presented as mean ± standard deviation (SD). Post-hoc multiple comparisons were performed using the Games-Howell method, which does not assume equal variances. Different superscript letters within the same row indicate significant differences among groups at *p*<0.05; groups sharing the same superscript letter are not significantly different. For simplicity, consecutive letter ranges (e.g., a-e, b-e) are used to represent extended strings of superscript letters.

**Table S5**. Climatic factors at the sampling locations

| Sample | Bio1 | Bio2 | Bio3 | Bio4 | Bio5 | Bio6 | Bio7 | Bio12 | Bio13 | Bio14 | Bio15 |
| --- | --- | --- | --- | --- | --- | --- | --- | --- | --- | --- | --- |
| F6 | 2.81 | 13.06 | 26.33 | 1333.27 | 25.00 | -24.60 | 49.60 | 751.00 | 183.00 | 8.00 | 96.01 |
| S4 | 11.85 | 6.43 | 20.36 | 909.35 | 26.80 | -4.80 | 31.60 | 712.00 | 175.00 | 11.00 | 92.10 |
| Y3 | 17.22 | 9.08 | 43.67 | 465.64 | 26.20 | 5.40 | 20.80 | 1401.00 | 317.00 | 10.00 | 88.22 |
| C1 | 7.52 | 13.28 | 29.07 | 1244.11 | 28.60 | -17.10 | 45.70 | 378.00 | 112.00 | 1.00 | 113.42 |

**Note**: The definitions of the bioclimatic variables are as follows: Bio1, Annual Mean Temperature，℃; Bio2, Mean Diurnal Range, ℃; Bio3, Isothermality, %; Bio4，Temperature Seasonality, ℃*100; Bio5, Max Temperature of Warmest Month, ℃; Bio6, Min Temperature of Coldest Month, ℃; Bio7, Temperature Annual Range, ℃; Bio12, Annual Precipitation, mm; Bio13, Precipitation of Wettest Month, mm; Bio14, Precipitation of Driest Month, mm; Bio15, Precipitation Seasonality, %).

**Table S6**. PERMANOVA of bacterial communities across ecological niches of different medicinal plants based on Unweighted Unifrac distance

| Group 1 | Group 2 | Sample size | Permutations | pseudo-F | p-value | q-value |
| --- | --- | --- | --- | --- | --- | --- |
| C1CK | C1BS | 10 | 999 | 1.66 | 0.01 | 0.01 |
| C1CK | C1RS | 10 | 999 | 2.12 | 0.01 | 0.01 |
| C1CK | C1SS | 10 | 999 | 2.84 | 0.01 | 0.01 |
| C1BS | C1RS | 10 | 999 | 1.28 | 0.02 | 0.02 |
| C1BS | C1SS | 10 | 999 | 2.06 | 0.02 | 0.02 |
| C1RS | C1SS | 10 | 999 | 1.69 | 0.01 | 0.01 |
| F6CK | F6BS | 10 | 999 | 1.19 | 0.01 | 0.01 |
| F6CK | F6RS | 10 | 999 | 1.47 | 0.01 | 0.01 |
| F6CK | F6SS | 10 | 999 | 2.00 | 0.01 | 0.01 |
| F6BS | F6RS | 10 | 999 | 1.28 | 0.01 | 0.01 |
| F6BS | F6SS | 10 | 999 | 1.78 | 0.01 | 0.01 |
| F6RS | F6SS | 10 | 999 | 1.44 | 0.01 | 0.01 |
| S4CK | S4BS | 10 | 999 | 2.03 | 0.00 | 0.01 |
| S4CK | S4RS | 10 | 999 | 2.67 | 0.01 | 0.02 |
| S4CK | S4SS | 10 | 999 | 2.91 | 0.01 | 0.01 |
| S4BS | S4RS | 10 | 999 | 1.42 | 0.04 | 0.04 |
| S4BS | S4SS | 10 | 999 | 2.15 | 0.01 | 0.01 |
| S4RS | S4SS | 10 | 999 | 1.54 | 0.03 | 0.03 |
| Y3CK | Y3BS | 10 | 999 | 2.69 | 0.01 | 0.01 |
| Y3CK | Y3RS | 10 | 999 | 4.04 | 0.01 | 0.01 |
| Y3CK | Y3SS | 10 | 999 | 3.04 | 0.01 | 0.01 |
| Y3BS | Y3RS | 10 | 999 | 1.83 | 0.01 | 0.01 |
| Y3BS | Y3SS | 10 | 999 | 1.46 | 0.01 | 0.01 |
| Y3RS | Y3SS | 10 | 999 | 1.95 | 0.01 | 0.01 |

**Note**: The PERMANOVA was performed using the QIIME 2 command "qiime diversity beta-group-significance". The reported *q*-values are p-values corrected for multiple testing, which provide a more reliable reference. A *q*-value < 0.05 indicates a significant difference in microbial community structure between groups.

**Table S7**. PERMANOVA of fungal communities across ecological niches of different medicinal plants based on Unweighted Unifrac distance

| Group 1 | Group 2 | Sample size | Permutations | pseudo-F | p-value | q-value |
| --- | --- | --- | --- | --- | --- | --- |
| C1CK | C1BS | 10 | 999 | 2.25 | 0.01 | 0.01 |
| C1CK | C1RS | 10 | 999 | 2.75 | 0.01 | 0.01 |
| C1CK | C1SS | 10 | 999 | 2.80 | 0.01 | 0.01 |
| C1BS | C1RS | 10 | 999 | 1.92 | 0.01 | 0.01 |
| C1BS | C1SS | 10 | 999 | 1.92 | 0.01 | 0.01 |
| C1RS | C1SS | 10 | 999 | 1.86 | 0.01 | 0.01 |
| F6CK | F6BS | 10 | 999 | 1.86 | 0.01 | 0.01 |
| F6CK | F6RS | 10 | 999 | 2.48 | 0.01 | 0.01 |
| F6CK | F6SS | 10 | 999 | 2.43 | 0.01 | 0.01 |
| F6BS | F6RS | 10 | 999 | 1.69 | 0.01 | 0.01 |
| F6BS | F6SS | 10 | 999 | 1.75 | 0.01 | 0.01 |
| F6RS | F6SS | 10 | 999 | 1.58 | 0.01 | 0.01 |
| S4CK | S4BS | 10 | 999 | 4.09 | 0.01 | 0.01 |
| S4CK | S4RS | 10 | 999 | 4.41 | 0.01 | 0.01 |
| S4CK | S4SS | 10 | 999 | 5.12 | 0.01 | 0.01 |
| S4BS | S4RS | 10 | 999 | 2.78 | 0.01 | 0.01 |
| S4BS | S4SS | 10 | 999 | 2.93 | 0.01 | 0.01 |
| S4RS | S4SS | 10 | 999 | 1.40 | 0.01 | 0.01 |
| Y3CK | Y3BS | 10 | 999 | 1.92 | 0.03 | 0.03 |
| Y3CK | Y3RS | 10 | 999 | 3.47 | 0.01 | 0.01 |
| Y3CK | Y3SS | 10 | 999 | 2.61 | 0.01 | 0.01 |
| Y3BS | Y3RS | 10 | 999 | 1.98 | 0.01 | 0.01 |
| Y3BS | Y3SS | 10 | 999 | 1.56 | 0.01 | 0.01 |
| Y3RS | Y3SS | 10 | 999 | 1.35 | 0.01 | 0.01 |

**Note**: The PERMANOVA was performed using the QIIME 2 command "qiime diversity beta-group-significance". The reported *q*-values are p-values corrected for multiple testing, which provide a more reliable reference. A *q*-value < 0.05 indicates a significant difference in microbial community structure between groups.

**Fig. S1. Microbial community differentiation between cultivated and uncultivated soils based on Unweighted UniFrac distance
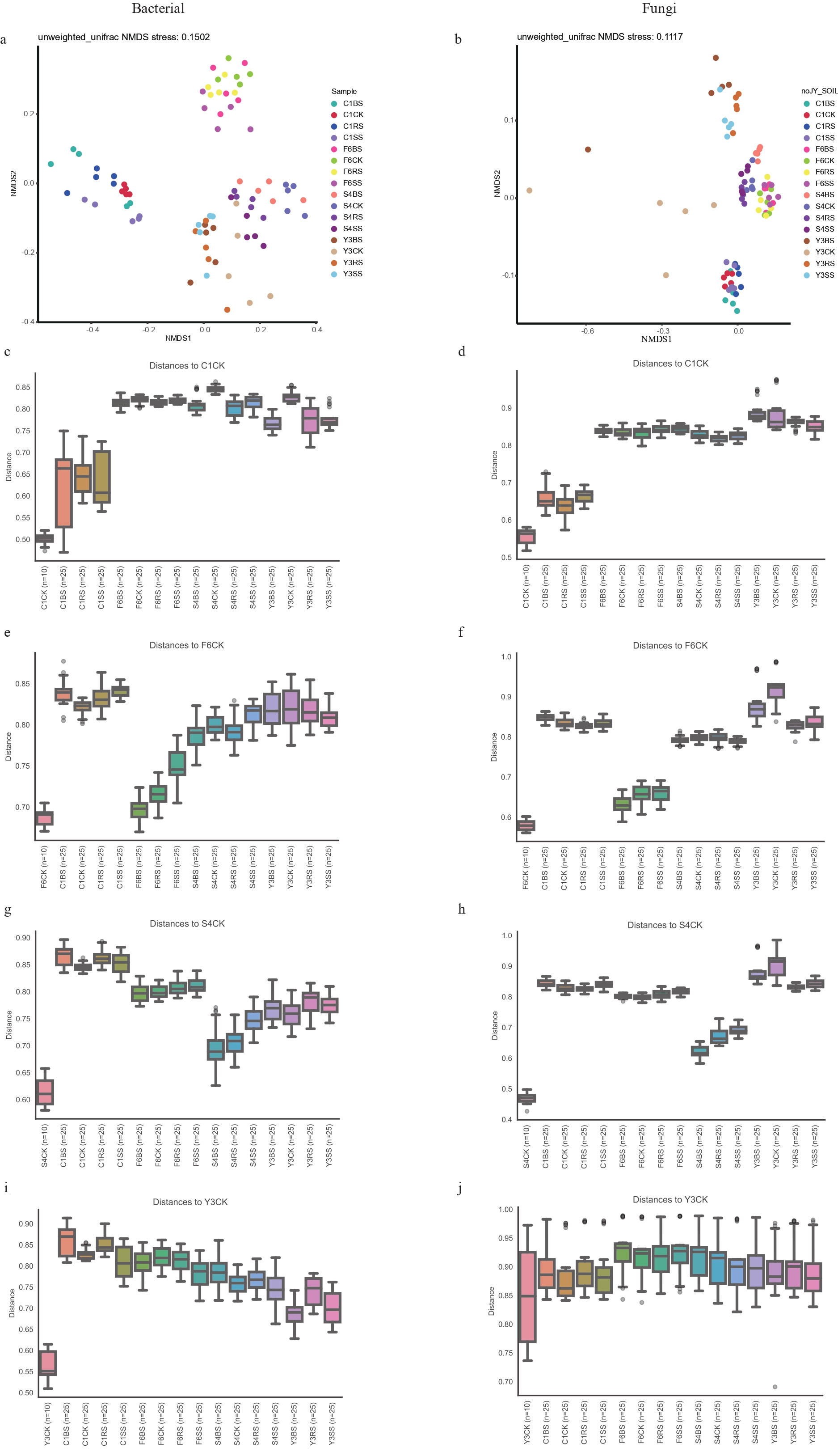
.** (a, b) NMDS ordination based on Unweighted Unifrac distance for bacterial and fungal communities, respectively. (c-j) Boxplots display inter-group comparisons: the horizontal axis represents group information, with the number in parentheses (n) indicating the number of pairwise comparisons between groups; the vertical axis represents the Unweighted Unifrac distance between samples. The number of boxplots corresponds to the number of groups. The plots shown here specifically illustrate the distances between cultivated soils and their adjacent uncultivated soils.

**

Fig. S2 Significance analysis of dominant soil phyla across different ecological regions of four plant species**. (a-g) represent dominant bacterial phyla, and (h-k) represent dominant fungal phyla, respectively. Welch's ANOVA was used for analysis; bars sharing the same letter are not significantly different (*p* < 0.05).

**Table S8** Topological properties of co-occurrence networks across different groups

| sample | Microbiota | Mod | ACC | Diameter | Density | AD |
| --- | --- | --- | --- | --- | --- | --- |
| C1BS | Bacteria | 0.942 | 1 | 1 | 0.015 | 7.111 |
|  | Fungi | 0.806 | 1 | 1 | 0.052 | 7.603 |
| C1CK | Bacteria | 0.919 | 1 | 1 | 0.016 | 7.429 |
|  | Fungi | 0.808 | 1 | 1 | 0.045 | 7.031 |
| C1RS | Bacteria | 0.912 | 1 | 1 | 0.016 | 7.811 |
|  | Fungi | 0.829 | 1 | 1 | 0.046 | 7.754 |
| C1SS | Bacteria | 0.856 | 1 | 1 | 0.023 | 10.741 |
|  | Fungi | 0.815 | 1 | 1 | 0.046 | 7.861 |
| F6BS | Bacteria | 0.842 | 1 | 1 | 0..032 | 14.768 |
|  | Fungi | 0.834 | 1 | 1 | 0.039 | 10.282 |
| F6CK | Bacteria | 0.75 | 1 | 1 | 0.033 | 15.287 |
|  | Fungi | 0.842 | 1 | 1 | 0.039 | 9.592 |
| F6RS | Bacteria | 0.847 | 1 | 1 | 0.028 | 12.129 |
|  | Fungi | 0.824 | 1 | 1 | 0.035 | 8.706 |
| F6SS | Bacteria | 0.893 | 1 | 1 | 0.028 | 11.254 |
|  | Fungi | 0.865 | 1 | 1 | 0.029 | 8.065 |
| S4BS | Bacteria | 0.795 | 1 | 1 | 0.029 | 13.532 |
|  | Fungi | 0.853 | 1 | 1 | 0.031 | 6.716 |
| S4CK | Bacteria | 0.805 | 1 | 1 | 0..042 | 16.513 |
|  | Fungi | 0.827 | 1 | 1 | 0.035 | 5.732 |
| S4RS | Bacteria | 0.666 | 1 | 1 | 0.027 | 12.504 |
|  | Fungi | 0.798 | 1 | 1 | 0.044 | 7.978 |
| S4SS | Bacteria | 0.816 | 1 | 1 | 0..034 | 15.419 |
|  | Fungi | 0.851 | 1 | 1 | 0.037 | 5.939 |
| Y3BS | Bacteria | 0.801 | 1 | 1 | 0.032 | 14.864 |
|  | Fungi | 0.664 | 1 | 1 | 0.127 | 12.042 |
| Y3CK | Bacteria | 0.79 | 1 | 1 | 0.049 | 22..716 |
|  | Fungi | 0.321 | 1 | 1 | 0.204 | 23.607 |
| Y3RS | Bacteria | 0.946 | 1 | 1 | 0.016 | 7.363 |
|  | Fungi | 0.816 | 1 | 1 | 0.057 | 8.014 |
| Y3SS | Bacteria | 0.899 | 1 | 1 | 0.023 | 10.549 |
|  | Fungi | 0.78 | 1 | 1 | 0.077 | 12.051 |

**Note**: Mol, ACC, and AD are the abbreviations for Modularity, Average Clustering Coefficient, and Average Degree, respectively.

**Table S9**. Analysis of structural heterogeneity in microbial co-occurrence networks based on module proportion

| sample | Microbiota | SD | Mean | CV | G | E |
| --- | --- | --- | --- | --- | --- | --- |
| C1BS | Bacteria | 0.01 | 0.03 | 0.28 | 0.78 | 0.42 |
|  | Fungi | 0.04 | 0.07 | 0.51 | 0.60 | 0.69 |
| C1CK | Bacteria | 0.01 | 0.03 | 0.43 | 0.80 | 0.42 |
|  | Fungi | 0.03 | 0.06 | 0.53 | 0.63 | 0.65 |
| C1RS | Bacteria | 0.01 | 0.04 | 0.36 | 0.77 | 0.45 |
|  | Fungi | 0.03 | 0.07 | 0.50 | 0.62 | 0.66 |
| C1SS | Bacteria | 0.02 | 0.04 | 0.45 | 0.74 | 0.50 |
|  | Fungi | 0.04 | 0.06 | 0.57 | 0.65 | 0.64 |
| F6BS | Bacteria | 0.03 | 0.05 | 0.53 | 0.70 | 0.57 |
|  | Fungi | 0.03 | 0.06 | 0.54 | 0.66 | 0.61 |
| F6CK | Bacteria | 0.03 | 0.05 | 0.68 | 0.75 | 0.53 |
|  | Fungi | 0.03 | 0.06 | 0.51 | 0.65 | 0.62 |
| F6RS | Bacteria | 0.03 | 0.05 | 0.53 | 0.73 | 0.53 |
|  | Fungi | 0.03 | 0.06 | 0.50 | 0.68 | 0.59 |
| F6SS | Bacteria | 0.02 | 0.05 | 0.31 | 0.66 | 0.58 |
|  | Fungi | 0.02 | 0.05 | 0.41 | 0.68 | 0.58 |
| S4BS | Bacteria | 0.03 | 0.05 | 0.62 | 0.75 | 0.52 |
|  | Fungi | 0.03 | 0.05 | 0.48 | 0.70 | 0.57 |
| S4CK | Bacteria | 0.03 | 0.06 | 0.50 | 0.64 | 0.64 |
|  | Fungi | 0.03 | 0.06 | 0.51 | 0.68 | 0.60 |
| S4RS | Bacteria | 0.03 | 0.04 | 0.82 | 0.79 | 0.46 |
|  | Fungi | 0.03 | 0.06 | 0.53 | 0.64 | 0.64 |
| S4SS | Bacteria | 0.03 | 0.05 | 0.52 | 0.68 | 0.58 |
|  | Fungi | 0.02 | 0.06 | 0.41 | 0.63 | 0.63 |
| Y3BS | Bacteria | 0.02 | 0.08 | 0.23 | 0.43 | 0.78 |
|  | Fungi | 0.08 | 0.10 | 0.77 | 0.52 | 0.78 |
| Y3CK | Bacteria | 0.03 | 0.07 | 0.48 | 0.59 | 0.68 |
|  | Fungi | 0.12 | 0.11 | 1.13 | 0.57 | 0.73 |
| Y3RS | Bacteria | 0.01 | 0.03 | 0.23 | 0.77 | 0.43 |
|  | Fungi | 0.04 | 0.07 | 0.58 | 0.60 | 0.68 |
| Y3SS | Bacteria | 0.02 | 0.04 | 0.36 | 0.72 | 0.52 |
|  | Fungi | 0.05 | 0.08 | 0.63 | 0.56 | 0.73 |

**Note**: SD: Standard Deviation; Mean: Mean; CV: Coefficient of Variation; G: Gini coefficient; E: Shannon's Evenness Index. All metrics were calculated based on the proportion of Module 1-8.

**
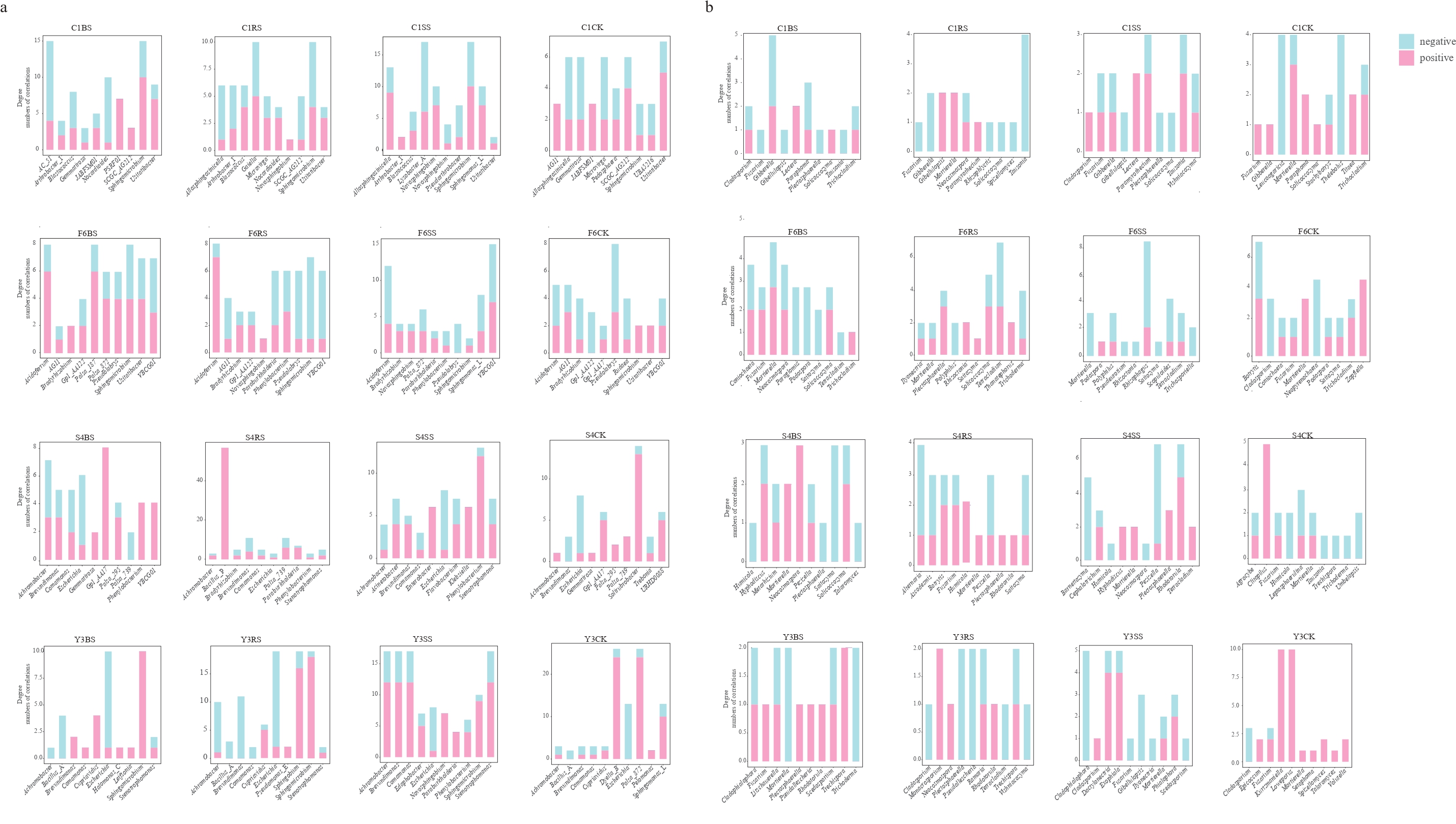
**

**Fig. S3**. Degree and interaction types of the top 10 nodes (at the genus level) in the soil microbial co-occurrence networks from the root zones of four medicinal plants. (a) Bacterial genera. (b) Fungal genera. The stacked proportions in the bars represent the composition of positive (red) and negative (blue) interactions.

**

Fig. S4**. Differential analysis of major predicted metabolic pathways in microbial community function. (a-f) Top six functions predicted from bacterial community data; (g-k) Top five functions predicted from fungal community data. Differences among groups were tested by Welch's one-way analysis of variance (ANOVA) at *p* < 0.05.

**List of Abbreviations**

CCP: Continuous Cropping Problem

*P. ginseng*: *Panax ginseng*

*P. quinquefolius*: *Panax quinquefolius*

*P. notoginseng*: *Panax notoginseng*

*A. bidentata*: *Achyranthes bidentata*

F6RS: Rhizosphere Soil of *Panax ginseng*

F6SS: Rhizoplane Soil of *Panax ginseng*

F6BS: Bulk Soil of *Panax ginseng*

F6CK: Uncultivated Control Soil of *Panax ginseng*

S4RS: Rhizosphere Soil of *Panax quinquefolius*

S4SS: Rhizoplane Soil of *Panax quinquefolius*

S4BS: Bulk Soil of *Panax quinquefolius*

S4CK: Uncultivated Control Soil of *Panax quinquefolius*

Y3RS: Rhizosphere Soil of *Panax notoginseng*

Y3SS: Rhizoplane Soil of *Panax notoginseng*

Y3BS: Bulk Soil of *Panax notoginseng*

Y3CK: Uncultivated Control Soil of *Panax notoginseng*

C1RS: Rhizosphere Soil of *Achyranthes bidentata*

C1SS: Rhizoplane Soil of *Achyranthes bidentata*

C1BS: Bulk Soil of *Achyranthes bidentata*

C1CK: Uncultivated Control Soil of *Achyranthes bidentata*

WC: Water content

OM: Organic Matter

TN: Total nitrogen

TP : Total phosphorus

TK : Total potassium

AN : Available nitrogen

AP : Available phosphorus

AK: Available potassium

NO_3_^-^-N : Nitrate nitrogen

NH_4_^+^-N: Ammonium nitrogen

S-URE ：Urease enzyme activity

S-ACP : Acid phosphatase enzyme activity

S-SUC : Sucrase enzyme activity

S-CAT : Catalase enzyme activity

S-β-GC: β-glucosidase enzyme activity

S-NAR : Nitrate reductase enzyme activity

S-NIR : Nitrite reductase enzyme activity

Bio1: Annual Mean Temperature

Bio2: Mean Diurnal Range

Bio3: Isothermality

Bio4: Temperature Seasonality

Bio5: Max Temperature of Warmest Month

Bio6, Min Temperature of Coldest Month

Bio7, Temperature Annual Range

Bio12: Annual Precipitation

Bio13: Precipitation of Wettest Month

Bio14: Precipitation of Driest Month

Bio15: Precipitation Seasonality
